# Pioneer-factor activity requires stable chromatin occupancy mediated by both sequence-specific binding and disordered protein domains

**DOI:** 10.1126/sciadv.aef2244

**Published:** 2026-06-19

**Authors:** Meghan M. Freund, F. Javier deHaro-Arbona, Sarah Baloul, Ali Torhorst, Charalambos Roussos, Abby J. Ruffridge, Andrew Q. Rashoff, Ryen Hazzard, Peter W. Lewis, Sarah J. Bray, Melissa M. Harrison

**Affiliations:** ^1^ Department of Biomolecular Chemistry, University of Wisconsin-Madison, Madison, WI, USA.; ^2^ Department of Physiology, Development, and Neuroscience, University of Cambridge, Cambridge, UK.

## Abstract

Pioneer transcription factors overcome the restrictive barrier imposed by chromatin to drive cell-fate specification, yet how their domains collectively support this activity remains unclear. Here, we use the deeply conserved pioneer factor Grainy head to define the protein-intrinsic features that govern pioneering activity. By integrating biochemistry, genomics, and quantitative live-cell imaging, we determined that both the conserved DNA-binding domain and the extended, intrinsically disordered N terminus are required for the stable chromatin occupancy that supports access to closed chromatin and the induction of chromatin accessibility. The disordered N terminus supports pioneer activity through interactions that do not rely on strict amino acid sequence but instead on overall composition. While our results show that pioneering activity depends on the combinatorial contributions of structured and disordered domains, mitotic retention depends solely on sequence-specific DNA binding. These results support stable chromatin occupancy mediated by multiple protein domains as necessary for pioneering function and that this is separable from the mechanisms required for mitotic retention.

## Teaser

Independent of sequence, disordered regions promote the stable chromatin occupancy required for pioneer-factor activity.

## INTRODUCTION

The gene-expression programs that control cellular identity are driven by transcription factors that recognize specific DNA sequence motifs. However, transcription-factor binding is constrained by the packaging of DNA into chromatin, which restricts access to much of the genome (*1*). A subset of transcription factors, termed pioneer factors, overcome this barrier by binding to nucleosomal DNA, promoting local chromatin accessibility, and enabling broad transcriptional reprogramming events (*2*–*5*). These features allow pioneer factors to function as drivers of cell-fate specification. A set of characteristics has been used to define pioneer factors, including binding nucleosomes in vitro; binding closed, nucleosome-occupied chromatin in cells; and promoting chromatin accessibility (*6*). In addition, a subset of pioneer factors are retained on mitotic chromosomes and implicated in the rapid reinitiation of transcription following cell division (*7*, *8*). Nonetheless, these properties are not shared by all pioneer factors, and the mechanistic details by which individual factors engage and open chromatin differ (*6*). To address how pioneer factors uniquely bind the genome, it is necessary to systematically test the relationship between these defining properties.

Structured DNA-binding domains (DBDs) provide sequence specificity for transcription factors and, in many cases, are sufficient for nucleosome binding by pioneer factors in vitro (*9*–*13*). However, recent in vivo assays demonstrate that DBDs alone cannot explain the full spectrum of pioneering activity (*14*, *15*). Non-DBDs contribute to chromatin engagement (*16*–*21*) and, in some cases, can direct binding on their own (*16*). Pioneer factors contain diverse non-DBDs that differ widely in amino acid composition, yet frequently include intrinsically disordered regions (IDRs). These IDRs have been proposed to facilitate target search, stabilize chromatin interactions, recruit cofactors, and drive condensate formation (*20*, *22*–*29*). Despite the emerging roles of IDRs, they lack conserved sequence motifs, making the mechanism by which they support pioneering unclear.

Grainy head (Grh) is an essential and deeply conserved transcription factor that drives expression of genes that promote epithelial cell fate in organisms ranging from worms to humans (*30*–*39*). Grh was initially identified in *Drosophila melanogaster*, where it is encoded by a single gene (*30*, *40*–*42*). By contrast, mammals have three Grh-like proteins (GRHL1-3) whose dysregulation is associated with numerous epithelial cancers (*34*, *36*, *43*–*45*). Grh proteins from both flies and mice exhibit key features of pioneer factors: Loss of Grh reduces chromatin accessibility, whereas ectopic expression promotes accessibility at previously closed sites (*14*, *46*–*48*). The structure of Grh is shared across species and contains a highly conserved DBD and dimerization domain (*32*, *34*, *49*). In all organisms studied, Grh binds the same DNA motif, reflecting the strong conservation of the Grh DBD (*33*, *36*, *37*). By contrast, the N terminus is poorly conserved and shows notable divergence in length, sequence, and predicted structure (*50*). Because of the shared roles of GRHL proteins in pioneering and their impact on human health, we use the simplified *Drosophila* system to determine how both ordered and disordered protein domains individually contribute to multiple, defining features of pioneer factors. By integrating biochemical, genomic, and imaging assays, we reveal that the stable chromatin occupancy required for pioneering activity depends on sequence-specific DNA binding and additional protein domains, which can vary in sequence and structure. By contrast, mitotic retention relies solely on sequence-specific DNA binding.

## RESULTS

### Grh has features of a pioneer transcription factor

**Fig. 1.**
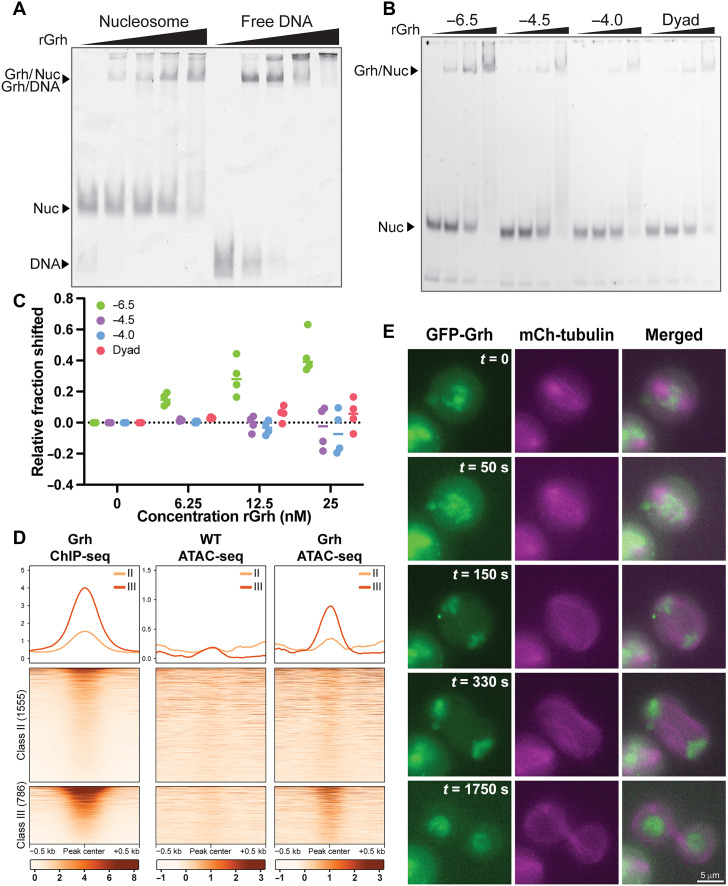
Grh has features of a pioneer transcription factor. (**A**) EMSA with increasing concentrations of recombinant Grh (rGrh) (0 to 50 nM) incubated with Cy5-labeled free or nucleosomal (Nuc) DNA. (**B**) EMSA with increasing concentrations of rGrh (0 to 25 nM) incubated with Cy5-labeled Widom 601 nucleosomes with the canonical Grh motif positioned at SHLs −6.5, −4.5, −4.0, or 0 (dyad). (**C**) Relative fraction of Grh-bound nucleosomes containing the respective Grh motif as compared to nucleosomes lacking the Grh motif, measured by comparing the proportion shifted in EMSAs shown in (B) and fig. S1C and three additional experiments. (**D**) Heatmaps and metaplots of binding by Grh (ChIP-seq) and chromatin accessibility (ATAC-seq) in wild-type (WT) S2 cells or those in which Grh is induced. Classes are defined by Gibson *et al.* (*14*). (**E**) Images of S2 cells expressing GFP-Grh (green) and mCherry-tubulin (magenta) over mitosis. *t*, time in seconds relative to the initial image (metaphase).

To find how distinct features of pioneer factors relate to each other, we first determined the essential pioneering features of Grh. Despite being identified as a pioneer factor, whether Grh can directly engage nucleosomal DNA has not been tested directly (*14*, *46*–*48*). Therefore, we reconstituted nucleosomes containing an endogenous target sequence and tested binding by full-length recombinant Grh in vitro. We identified a genomic locus in closed chromatin that contains a canonical Grh-binding motif and becomes accessible upon Grh expression and binding in *Drosophila* Schneider 2 (S2) cells (fig. S1A) (*14*). We generated nucleosomes using a 159–base pair (bp) fragment surrounding the Grh motif. In electrophoretic mobility shift assays (EMSAs), full-length recombinant Grh bound to both free and nucleosomal DNA (Fig. 1A). Together, these data establish that Grh directly interacts in vitro with nucleosomal DNA, a defining feature of pioneer factors.

Pioneer factors vary in their recognition of nucleosomal motifs, with some binding at the dyad, some at the edge, and others at sites in between (*12*, *51*, *52*). To test the motif position preference for Grh, we took advantage of the well-positioned Widom 601 nucleosome, allowing us to insert the Grh motif at defined positions around the nucleosome axis (*53*). We placed the motif at the edge [super helical location (SHL) −6.5], internal positions (SHL −4.5 and −4.0, solvent exposed and buried, respectively), and the dyad (SHL 0) and assayed binding by recombinant Grh (fig. S1B). Motif-specific binding was observed when the Grh motif was positioned at SHL −6.5, whereas binding at internal positions (SHL −4.5 and −4.0) was not enriched above binding to nucleosomes lacking the motif (Fig. 1, B and C, and fig. S1, C and D). Grh bound to nucleosomes containing the motif at the dyad slightly better than to nucleosomes lacking the motif but only shifted a small fraction of the total nucleosomes (Fig. 1C and fig. S1D). This binding preference resembles that reported for other pioneer factors and likely reflects the dynamic nature of DNA wrapping at the nucleosome entry/exit site (*51*, *54*, *55*).

To examine pioneering activity in a cellular context, we ectopically expressed Grh at physiological levels in S2 cells, which do not endogenously express Grh, and assayed genome-wide binding using chromatin immunoprecipitation coupled with sequencing (ChIP-seq) and chromatin accessibility using assay for transposase-accessible chromatin coupled with sequencing (ATAC-seq) (*14*). As reported previously, Grh-bound sites fall into three categories distinguished by their accessibility before and after Grh binding (*14*). Class I sites are accessible regions that Grh occupies without altering accessibility and are sites of promiscuous, nonspecific binding (fig. S2) (*14*). Class II and class III sites are inaccessible prior to binding and reflect the ability of Grh to function as a pioneer factor. These classes differ in their response to Grh binding with class II sites remaining inaccessible and class III sites becoming accessible (Fig. 1D and fig. S2). These data demonstrate that, at a subset of loci, Grh functions as a pioneer factor by binding to closed chromatin and promoting accessibility.

Another feature commonly associated with pioneer factors is mitotic retention. During mitosis, most transcription factors are not retained on chromatin with only a subset remaining bound (mitotically retained) (*56*–*58*). These mitotically retained factors are thought to facilitate rapid reactivation of gene expression after mitosis (*7*, *59*–*62*). Although the mechanisms of mitotic retention are not well understood, features that promote pioneer activity are thought to contribute to retention, as many pioneer factors are mitotically retained (*7*, *8*, *47*, *59*, *61*–*66*). Endogenous Grh is mitotically retained at euchromatin in the gastrulating embryo (*47*). We examined whether this feature is shared in S2 cells, a tractable cellular system. Using mCherry-tubulin to mark mitotic cells, we identified a clear association of transiently expressed green fluorescent protein (GFP)–Grh with mitotic chromosomes, recapitulating prior in vivo observations (Fig. 1E) (*47*). These results, along with previous data, demonstrate that Grh has multiple, defining features of pioneer factors and establish assays to test the contribution of various protein domains to these pioneering activities (*14*, *46*–*48*).

### The DBD of Grh and regions outside are required for nucleosome binding and opening chromatin

**Fig. 2.**
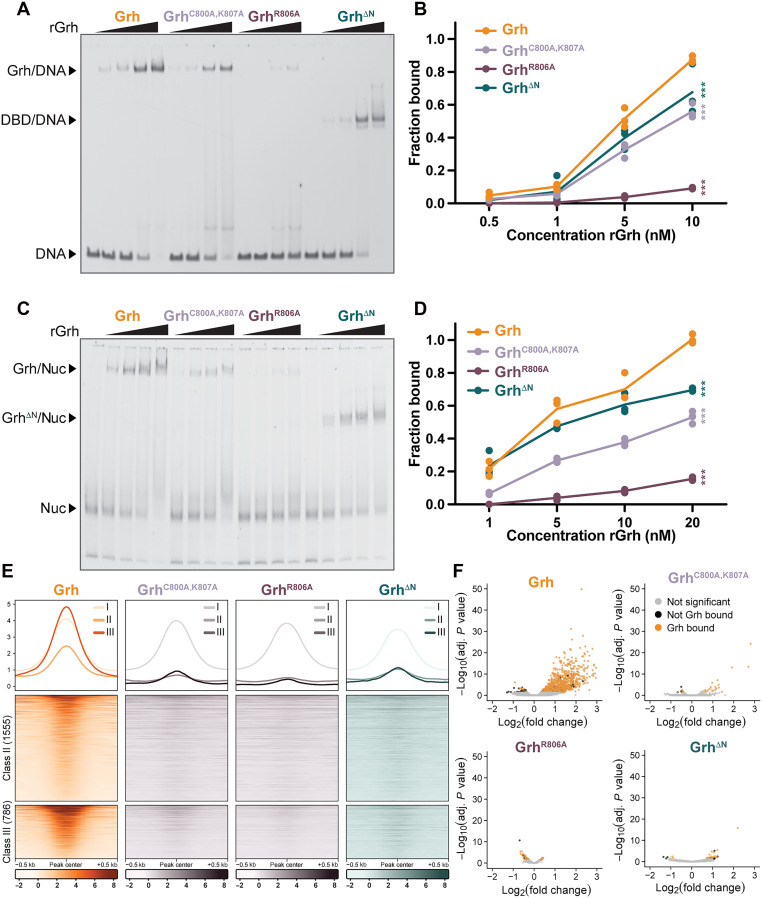
Grh requires the DBD and regions outside for binding nucleosomes and opening chromatin. (**A**) EMSA with increasing concentrations of rGrh, Grh^C800A,K807A^, Grh^R806A^, or Grh^ΔN^ (0 to 10 nM) incubated with Cy5-labeled DNA. (**B**) Quantification of the fraction of Grh-bound DNA, indicated by the proportion shifted in the EMSA shown in (A) and two additional experiments. Significance compared to rGrh was determined with a two-way analysis of variance (ANOVA) with post hoc Dunnett’s multiple comparisons test and, for simplicity, is shown for the highest concentration tested. ***P < 0.0005. (**C**) EMSA with increasing concentrations of rGrh, Grh^C800A,K807A^, Grh^R806A^, or Grh^ΔN^ (0 to 20 nM) incubated with Cy5-labeled nucleosomal DNA. (**D**) Quantification of the fraction of Grh-bound nucleosomes, indicated by the proportion shifted in the EMSA shown in (C) and two additional experiments. Significance compared to rGrh was determined with a two-way ANOVA with post hoc Dunnett’s multiple comparisons test and, for simplicity, is shown only for the highest concentration tested. ***P < 0.0005. (**E**) Heatmaps and metaplots of ChIP-seq for Grh, Grh^C800A,K807A^, Grh^R806A^, or Grh^ΔN^ induced in S2 cells. Class I shown in metaplots (and fig. S3D), and class II and III shown in both metaplots and heatmaps. Grh^ΔN^ data from Gibson *et al.* (*14*). (**F**) Volcano plots of changes in ATAC-seq signal upon expression of Grh, Grh^C800A,K807A^, Grh^R806A^, or Grh^ΔN^ as compared to uninduced controls. Grh^ΔN^ data from Gibson *et al.* (*14*). Regions bound by wild-type Grh, as defined by Gibson *et al.* (*14*), shown in orange.

Having established the core pioneering features of Grh, we next determined how the DBD and the disordered N terminus contributed to in vitro nucleosome binding and how this relates to the ability of Grh to promote chromatin accessibility in culture. To test the role of DNA binding, we leveraged the high degree of conservation in the DBD and the published structure of the mammalian Grh-like protein GRHL1 to identify residues that directly contact DNA (*67*). We generated purified full-length protein with mutations that are predicted to abrogate binding to either the phosphate backbone (Grh^C800A,K807A^) or the conserved guanine in the Grh motif (Grh^R806A^) (fig. S3A). Consistent with the role of these residues in promoting DNA binding, both DBD mutations reduced binding to free DNA, with Grh^R806A^ showing a stronger decrease than Grh^C800A,K807A^ (Fig. 2, A and B, and fig. S3B). To assess the influence of the large, disordered N terminus, we purified protein that corresponded to only the C-terminal DBD and dimerization domains (Grh^ΔN^). Grh^ΔN^ had decreased binding to free DNA relative to wild-type protein but retained some DNA-binding activity (Fig. 2, A and B, and fig. S3B). Because pioneering involves interactions with DNA that is wrapped around histones, we tested the capacity of each mutant protein to bind nucleosomes. Similar to binding to free DNA, both DBD mutants reduced nucleosome binding, with Grh^R806A^ showing the strongest decrease, and Grh^ΔN^ exhibiting a modest but statistically significant reduction relative to wild-type Grh (Fig. 2, C and D). These results indicate that in vitro nucleosome binding reflects the ability to bind free DNA and requires both the DBD and N-terminal domain, with a particular dependence on Arg^806^ (R806), which mediates sequence-specific DNA contacts.

To determine how binding to nucleosomes in vitro correlated with pioneering activity in cells, we expressed both DBD mutants and Grh^ΔN^ in S2 cells and assayed binding and chromatin accessibility. All three proteins retained binding to most class I sites, reflecting their ability to occupy accessible chromatin (Fig. 2E and fig. S3, C and D) (*14*). Both Grh^C800A,K807A^ and Grh^R806A^ had reduced binding to accessible sites containing the Grh motif, supporting the role of these residues in promoting sequence-specific Grh binding (Fig. 2E and fig. S3D). In contrast to their ability to bind to open chromatin, all three proteins had markedly decreased binding to closed chromatin (both class II and class III sites) (Fig. 2E) (*14*). Consistent with the loss of binding at inaccessible sites, these mutants failed to open chromatin, despite being expressed at levels higher than wild type (Fig. 2F and fig. S3, E and F) (*14*). Thus, while Grh^C800A,K807A^ and Grh^ΔN^ had modest effects on binding in vitro, the reduction in chromatin binding was much more pronounced when assayed in cells. We next evaluated the contributions of these domains to transcriptional activation using transient transfections of a luciferase reporter driven by the promoter for *homogentisate 1,2-dioxygenase* (*hgo*), an established Grh target containing the Grh motif (*68*). Both DBD mutants and Grh^ΔN^ failed to activate the reporter, indicating that the structured DBD and the disordered N terminus are required to promote transcription (fig. S4, A and B). By assaying the same constructs both in vitro and in cells, we demonstrate that mononucleosome binding only partially captures the requirements for pioneering and that pioneering activity requires both DNA binding and activities provided by the N terminus.

### Grh requires the DBD and regions outside for stable chromatin occupancy

**Fig. 3.**
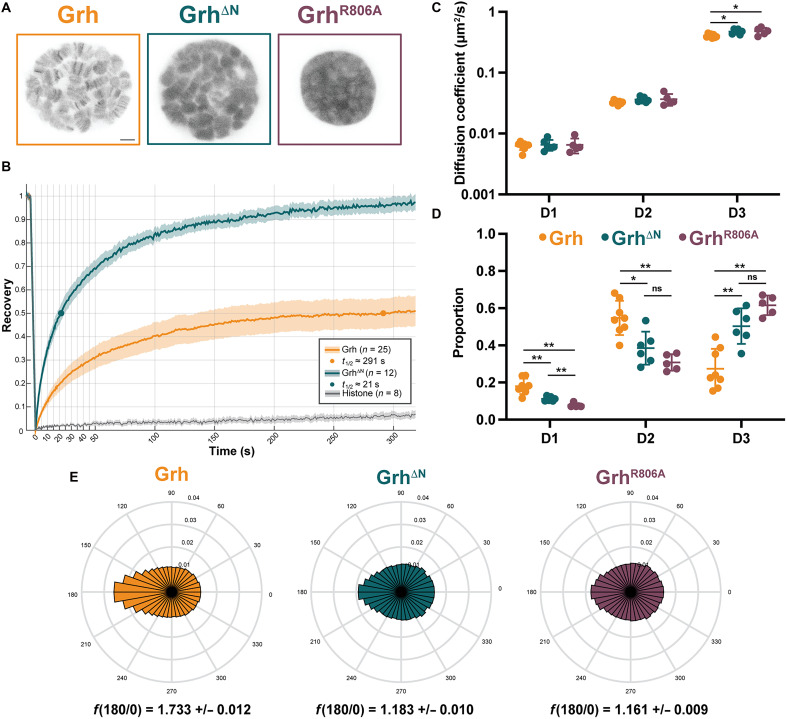
Grh requires the DBD and regions outside for stable chromatin occupancy. (**A**) Representative images of single nuclei of third instar larval salivary glands labeled with JF646 to visualize expression of Halo-tagged Grh, Grh^ΔN^, or Grh^R806A^. Scale bar, 5 μm. All images at the same magnification. (**B**) Recovery of Grh or Grh^ΔN^ molecules after photobleaching. Legend summarizes numbers of nuclei (*n*) and time to 50% recovery (*t*_1/2_). Shading indicates the standard error of the mean (SEM). (**C**) Average diffusion coefficients of three Grh populations as determined by vbSPT. Significance was assessed with two-sample *t* tests. *P < 0.05; unmarked comparisons showed no statistical difference. (**D**) Average proportion of Grh, Grh^R806A^, or Grh^ΔN^ molecules per nucleus found in each of the three states determined by vbSPT (Grh, *n* = 8 nuclei, 103,847 trajectories; Grh^R806A^, *n* = 5 nuclei, 39,946 trajectories; Grh^ΔN^, *n* = 6 nuclei, 50,109 trajectories). Significance was assessed with Mann-Whitney *U* tests. *P < 0.05; **P < 0.005. (**E**) Circular histograms of angle distributions calculated from D3 trajectories. Fold anisotropy *f*(180/0) ± standard deviation (SD) is noted for each sample. ns, not significant.

We hypothesized that the disconnect between mononucleosome binding and pioneering activity in cells might arise from differences in protein dynamics in vivo. Both EMSAs and ChIP-seq provide static views of protein binding. We therefore sought to use in vivo imaging to determine how the DBD and N terminus contribute to the dynamics of Grh binding. Because Grh binds DNA as a dimer, we expressed the mutants in a tissue that lacks endogenous protein to ensure that we were not assaying binding of mixed dimers (*32*, *49*). For this reason, we chose to express Halo-tagged Grh constructs in *Drosophila* salivary glands, which have large polytene chromosomes that can be easily visualized in intact nuclei and are ideal for live imaging of chromatin dynamics (*69*). To assay proteins that show a range of binding in our prior assays (Fig. 2), we expressed Halo-tagged wild-type Grh, Grh^R806A^, or Grh^ΔN^ and analyzed chromosomal occupancy following incubation with the Halo ligand Janelia Fluor 646 (JF646). Wild-type Grh was strongly chromosome-associated and was localized to multipole distinct bands, corresponding to individual genomic loci (Fig. 3A). Grh^ΔN^ was enriched on the chromosomes but with less clear localization to distinct bands than the full-length protein and with some protein remaining diffuse in the nucleoplasm (Fig. 3A). By contrast, Grh^R806A^ was present in the nucleoplasm but appeared to be largely excluded from chromosomes (Fig. 3A). We then performed fluorescence recovery after photobleaching (FRAP) to quantitatively assess the mobility of Grh on chromatin. We assayed wild-type Grh and Grh^ΔN^, focusing on the chromatin-bound fraction that was visualized through colocalization with the chromosomes. Grh^R806A^ was excluded from analysis as it was not detectably present on chromosomes. Wild-type Grh recovered slowly (*t*_1/2_ = 291 s), indicating a slow exchange of molecules, and never achieved full recovery within the imaging window, plateauing at 50%. This indicates that there is a substantial fraction of the protein that is essentially immobile over the time course of the experiment (Fig. 3B). Grh^ΔN^ recovered markedly faster (*t*_1/2_ = 21 s) and reached close to 100% recovery, indicating that the majority of chromosomally associated mutant proteins are rapidly exchanging. Hence, the N terminus is required for stable chromatin occupancy (Fig. 3B).

To further investigate the dynamics of Grh binding, we performed single-particle tracking (SPT) using sparse labeling of the Halo-tagged proteins. Molecules were imaged at 50 ms per frame, and their trajectories were tracked and subsequently segregated into three distinct diffusive states, using a variational Bayesian treatment of Hidden Markov Models (vbSPT) (*70*). The 50-ms frame rate biases the populations assayed toward less freely diffusing molecule by blurring out the fastest trajectories. The resulting states were D1, the slowest diffusing population likely corresponding to stably bound molecules; D2, a population whose diffusion coefficient is consistent with binding but is not as confined as D1; D3, the most mobile population, which is likely composed of transiently interacting molecules. Diffusion coefficients for each state were similar between wild-type Grh, Grh^R806A^, and Grh^ΔN^ populations, confirming that the same population characteristics were sampled under all conditions (Fig. 3C). In comparison to wild-type Grh, the proportions of Grh^R806A^ and Grh^ΔN^ molecules in the D1 and D2 state were considerably reduced, consistent with less stable chromatin engagement and with the lack of enrichment of Grh^R806A^ on chromosomes (Fig. 3, A and D). To further examine how the DBD and N terminus contribute to Grh chromatin recruitment, we measured the distribution of the angles within the D3 trajectories (see Materials and Methods). Any deviation of the angle distributions from uniform is indicative of behaviors that differ from Brownian motion, such that molecules with more confined behaviors indicative of searching behavior will have greater angular anisotropy. Wild-type Grh molecules had a high degree of angular anisotropy while both Grh^ΔN^ and Grh^R806A^ displayed reduced anisotropy, demonstrating less constrained movement and implying a decrease in searching behavior (Fig. 3E). Our quantitative assessment of how the DBD and N terminus contribute to the dynamics of Grh chromatin occupancy shows that DNA binding is essential for chromatin occupancy. By contrast, the disordered N terminus of Grh supports stable residence time and aids local searching but is not essential for transient chromatin association.

### The intrinsically disordered N terminus contributes to pioneering independent of amino acid sequence

**Fig. 4.**
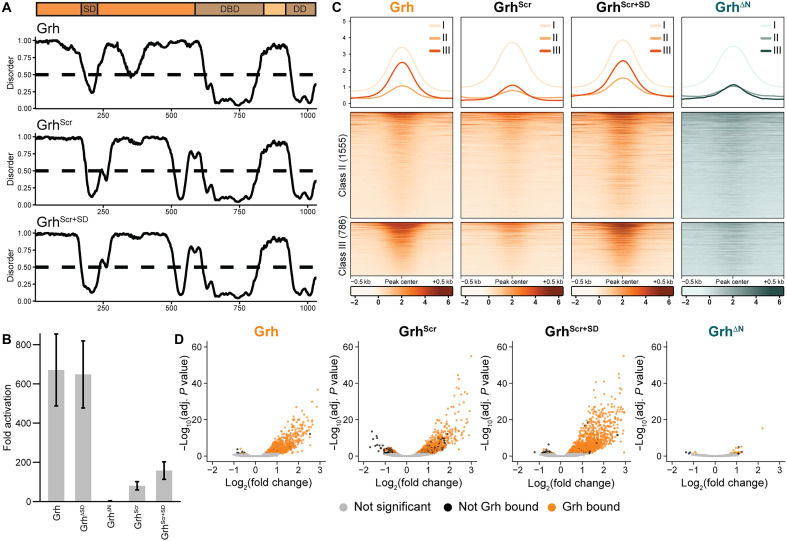
The intrinsically disordered Grh N terminus can contribute to pioneering independent of amino acid sequence. (**A**) Graph of predicted disorder score [from Metapredict; (*87*)], Grh (top), Grh^Scr^ (center), and Grh^Scr + SD^ (bottom). (**B**) Fold activation of the transiently transfected *hgo* luciferase reporter by either Grh, Grh^ΔSD^, Grh^ΔN^, Grh^Scr^, or Grh^Scr + SD^. *n* = 3, mean ± S.D. (**C**) Heatmaps and metaplots of ChIP-seq for Grh, Grh^Scr^, Grh^Scr + SD^, or Grh^ΔN^ expressed in S2 cells. Class I shown in metaplots (and fig. S5D), and class II and III shown in both metaplots and heatmaps. Data for Grh^ΔN^ are from Gibson *et al.* (*14*). (**D**) Volcano plots of changes in ATAC-seq signal upon expression of Grh, Grh^Scr^, or Grh^Scr + SD^ as compared to uninduced controls. Regions bound by wild-type Grh, as defined by Gibson *et al.* (*14*), shown in orange.

Given the critical role of the DBD in mediating DNA contacts, its necessity for pioneering activity was expected. By contrast, the mechanisms by which the disordered N terminus contributes to stable chromatin occupancy and pioneering activity were less clear. The N terminus is highly disordered apart from a short-structured domain (SD) that has previously been implicated in transcriptional activation (Fig. 4A) (*49*). To determine whether the SD was required for transcriptional activation in our assays, we tested whether Grh lacking this domain (Grh^ΔSD^) could promote expression from the *hgo* promoter. In contrast to expectations, deletion of the SD did not affect Grh-mediated transcriptional activation of a transiently transfected reporter (Fig. 4B and fig. S5A). Thus, we largely focused on the disordered region of Grh. Because intrinsically disordered domains are a common feature among eukaryotic transcription factors and have been associated with pioneering activity, we asked whether the importance of the N-terminal domain reflects specific-sequence elements (*16*, *18*, *22*, *71*). We generated a scrambled N-terminal mutant (Grh^Scr^) that preserves the overall amino acid composition and predicted disorder but disrupts the sequence, while leaving the C-terminal DBD and dimerization domain intact (Fig. 4A). Scrambling the N-terminal sequence did not substantially alter the distribution of charge or hydropathy (fig. S5B). For consistency, we selected a scrambled sequence that retained a patch of predicted order around the same size and location as the SD in the wild-type protein but with an entirely scrambled amino acid sequence.

We tested the ability of Grh^Scr^ to activate transcription using the transiently transfected Grh-responsive *hgo* reporter. Although the levels of Grh^Scr^ were consistently lower than those of wild-type Grh, the scrambled N terminus restored low levels of transcriptional activation activity relative to Grh^ΔN^ (Fig. 4B and fig. S5A). Because the ability to activate transcription does not reflect pioneering activity, we directly assessed chromatin binding and opening using ChIP-seq and ATAC-seq, respectively (Fig. 4, C and D, and fig. S5, C to E). While Grh^Scr^ occupies a similar number of class I binding sites as wild-type Grh (fig. S5D), it exhibited low ChIP-seq signal at inaccessible sites with only half of class III sites bound, similar to Grh^ΔN^ (Fig. 4C) (*14*). However, whereas Grh^ΔN^ failed to open chromatin, the addition of the scrambled N terminus allowed Grh^Scr^ to open chromatin (Fig. 4D and fig. S5E) (*14*). Although Grh^Scr^ was expressed at slightly higher levels than wild type, Grh^ΔN^ fails to open chromatin even when overexpressed, indicating that the disordered N terminus in Grh^Scr^ is sufficient to partially restore pioneering activity (fig. S5, C and E) (*14*). Reintroduction of the structured domain into the scrambled N terminus to generate Grh^Scr + SD^ improved the capacity of Grh to activate transcription, bind closed chromatin, and promote chromatin accessibility (Fig. 4, B to D, and fig. S5E). Thus, the SD sequence, rather than structure alone, promotes pioneer activity. However, the capacity of the scrambled N terminus to confer partial activity suggests that sequence-specific features are not strictly required, and other properties of the disordered region support some aspects of pioneering.

### Diverse N-terminal domains are sufficient for pioneering

**Fig. 5.**
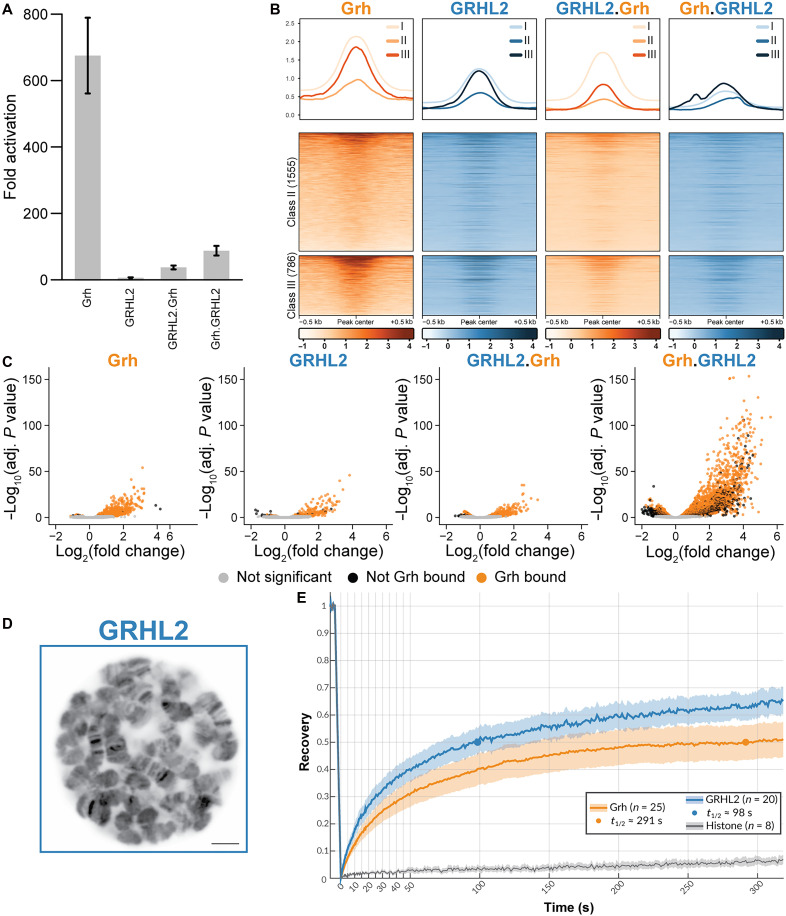
Diverse N-terminal domains are sufficient for pioneering. (**A**) Fold activation of the transiently transfected *hgo* luciferase reporter by either *Drosophila* Grh, human GRHL2, or chimeras (GRHL2.Grh and Grh.GRHL2). *n* = 3, mean ± S.D. (**B**) Heatmaps and metaplots of ChIP-seq for Grh, GRHL2, or chimeras expressed in S2 cells. Class I shown in metaplots (and fig. S6F), and class II and III shown in both metaplots and heatmaps. (**C**). Volcano plots of changes in ATAC-seq signal upon expression of *Drosophila* Grh, human GRHL2, or chimeras as compared to uninduced controls. Regions bound by wild-type Grh, as defined by Gibson *et al*. (*14*), shown in orange. (**D**) Representative image of single nuclei of third instar larval salivary glands labeled with JF646 to visualize expression of Halo-tagged GRHL2. Scale bar, 5 μm. (**E**) Recovery of Grh or GRHL2 molecules after photobleaching. Legend summarizes numbers of nuclei (*n*) and time to 50% recovery (*t*_1/2_). Shading indicates the SEM.

The requirement of the N-terminal domain for pioneering in *Drosophila* Grh contrasts with the widespread lack of conservation in this domain across species (*50*). While the mammalian GRHL family of proteins share the deeply conserved C-terminal DNA binding and dimerization domain, the N-terminal domain is truncated and less disordered than *Drosophila* Grh (fig. S6, A and B) (*35*–*37*). To expand on the properties of the N terminus that contribute to pioneering, we asked whether full-length human GRHL2 could function in *Drosophila* cells. Using the *hgo* promoter, we demonstrated that GRHL2 failed to activate transcription (Fig. 5A and fig. S6C). This lack of activity could reflect either a failure of GRHL2 to bind DNA in *Drosophila* cells or a lack of recruitment of cofactors needed to promote transcription. We therefore tested the pioneering capacity of GRHL2 using ChIP-seq and ATAC-seq on S2 cells expressing human GRHL2. GRHL2 was N-terminally tagged with a hemagglutinin (HA) epitope since the Grh antibody does not recognize human GRHL2. As a control, we similarly tagged *Drosophila* Grh with HA. A total of 99.6% of ChIP peaks identified with the HA antibody overlapped with peaks identified using antibodies recognizing Grh, demonstrating the specificity of the pulldowns (fig. S6D). In contrast to the failure of GRHL2 to promote gene expression from the *hgo* promoter, GRHL2 bound to 9533 loci, including substantial binding to both class II and class III regions of closed chromatin occupied by Grh (Fig. 5B and fig. S6, E and F). By comparing binding between Grh and GRHL2, we found that the shared sites had the highest ChIP signal for each protein and were enriched for the canonical Grh-binding motif (fig. S7, A and B). Sites bound uniquely by GRHL2 contained a more degenerate motif (fig. S7B). Because diverse non-DBD domains shape targeting to heterochromatin (*72*), we investigated whether sites bound by GRHL2 were enriched or depleted for H3K27me3. GRHL2 uniquely bound sites were depleted of H3K27me3 as compared to shared sites or those bound uniquely by Grh (fig. S7C), suggesting that the extended endogenous N terminus may help Grh bind a subset of loci in facultative heterochromatin. GRHL2 not only binds inaccessible sites, but ATAC-seq revealed that GRHL2 induces chromatin accessibility at these sites, although not to the extent of *Drosophila* Grh (Fig. 5C and fig. S6G). Thus, human GRHL2 can function as a pioneer factor in *Drosophila* cells, but this activity is not sufficient to promote gene expression.

Our quantitative imaging data suggested that the disordered N terminus stabilized chromatin occupancy and that this was correlated with pioneering activity. To determine whether the pioneering activity of human GRHL2 was similarly associated with stable chromatin occupancy, we expressed Halo-tagged GRHL2 in salivary gland nuclei. Supporting the ChIP-seq results that GRHL2 binds chromatin in *Drosophila*, we saw robust localization to the polytene chromosomes (Fig. 5D). FRAP demonstrated that human GRHL2 recovered with kinetics similar to *Drosophila* Grh with a substantial immobile fraction, reflecting stable chromatin occupancy (Fig. 5E). Together, the imaging and genomics analyses show that despite having a substantially shorter and more ordered N terminus than *Drosophila* Grh, human GRHL2 can stably bind closed chromatin and promote accessibility when expressed in *Drosophila* cells.

To further explore the individual contributions of the C terminus versus N terminus, we generated chimeric fusions between the human and *Drosophila* Grh proteins: GRHL2.Grh, in which the N-terminal domain of GRHL2 is fused to the *Drosophila* Grh DNA-binding and dimerization domains, and Grh.GRHL2, in which the *Drosophila* Grh N-terminal domain is fused to the GRHL2 DNA-binding and dimerization domains (fig. S6A). Both chimeric proteins weakly activate the *hgo* reporter, but not nearly to the extent of *Drosophila* Grh (Fig. 5A and fig. S6C). ChIP-seq and ATAC-seq demonstrated that the GRHL2.Grh chimera bound closed chromatin and promoted accessibility to a similar extent as GRHL2 (Fig. 5, B and C, and fig. S6, E to G). The Grh.GRHL2 fusion also bound closed chromatin to a similar extent as GRHL2 but promoted accessibility to a much greater degree (Fig. 5, B and C, and fig. S6G). Quantitative comparisons to *Drosophila* Grh were complicated by the relatively higher expression levels of GRHL2 and the fusion proteins (fig. S6E). Overall, these results indicate that while the human GRHL2 N terminus can confer pioneering activity to Grh, the extended *Drosophila* N terminus is most efficient at promoting chromatin accessibility in *Drosophila* cells.

### Mitotic retention of Grh requires DNA binding

**Fig. 6.**
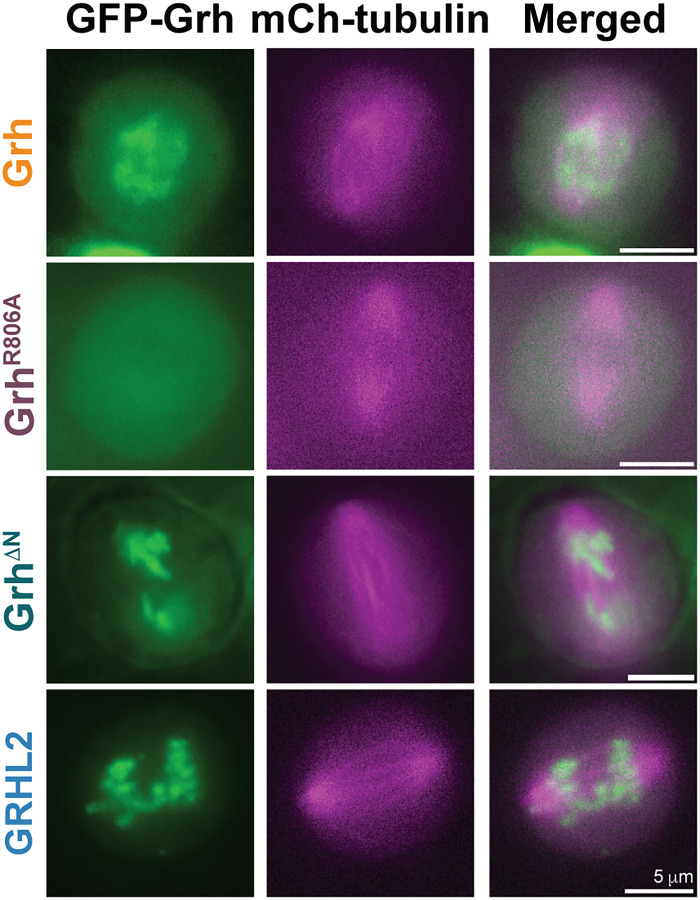
Mitotic retention of Grh requires only the C-terminal domains. Images of GFP-tagged Grh proteins (green) and mCherry-tubulin (magenta) at anaphase in S2 cells.

We further sought to connect pioneer activity with mitotic retention by testing the role of both the DBD and extended N terminus in retention of Grh on mitotic chromosomes. We examined mitotic retention of GFP-tagged Grh, Grh^ΔN^, Grh^R806A^, and GRHL2 in S2 cells, marking mitotic chromosomes with mCherry-tubulin. Grh^R806A^ was not mitotically retained, consistent with the importance of direct DNA contacts for pioneering and demonstrating that these contacts are also required for mitotic retention (Fig. 6 and fig. S8A). This lack of retention is not due to the change in charge, as substitution of the arginine with either lysine or histidine similarly abrogated mitotic retention (fig. S8B). Grh^ΔN^ showed robust retention, in stark contrast to the requirement of the N terminus for stable chromatin interactions and efficient pioneering activity (Fig. 6). GRHL2 was similarly retained (Fig. 6). Together, these results demonstrate that mitotic retention can be uncoupled from the stable chromatin occupancy required for pioneering activity but depends on DNA binding.

## DISCUSSION

Pioneer transcription factors are defined by their ability to engage nucleosomal DNA and initiate chromatin opening, enabling them to act at the top of gene-regulatory networks. Nonetheless, the essential defining features and how they contribute to the unique properties of pioneer factors remain controversial. Proteins with pioneering characteristics do not share DBD structures or sequences outside the DBD, adding further complexity to the challenge of defining this specialized class of transcription factors. To begin to address this complexity, we used multiple defining assays of pioneering function to identify the properties of the deeply conserved pioneer factor Grh. By doing so, we determined how these features relate to each other and how they rely on specific protein domains. Our assays reveal distinct requirements for pioneering and mitotic retention. Sequence-specific DNA binding, but not the multivalent interactions mediated by the disordered N terminus, is required for retention of Grh on mitotic chromatin. Thus, the set of intrinsic features enabling stable chromatin engagement during interphase is larger than that supporting chromatin associations during mitosis. This distinction is further supported by the fact that mitotic retention is not shared by a subset of pioneer factors, including Zelda and Ascl1 (*73*, *74*).

The DBD of Grh defines a family of transcription factors conserved from fungi to humans that bind to a shared DNA-sequence motif. In addition, DNA binding by Grh depends on dimerization mediated by a conserved dimerization domain (*32*, *49*). DBDs are classically thought of as the primary drivers of nucleosome targeting and, in vitro, are often sufficient for nucleosome binding (*9*, *11*, *12*). While Grh-family members were previously shown to bind and open closed chromatin, the ability to engage nucleosomes in vitro had not been investigated (*14*, *46*–*48*). Like many pioneer factors, Grh binds nucleosomes at the entry/exit site with affinities similar to that of free DNA. This interaction is largely mediated through sequence-specific binding by the C-terminal DBD and dimerization domains. However, the extended, disordered N terminus also contributes. This contrasts with the requirement for the N terminus for chromatin binding in cell culture, demonstrating that in vitro binding to mononucleosomes only partially reflects the requirements of pioneer factors to occupy the more complex chromatin environment in cells and is consistent with similar results for other pioneer factors (*15*). By combining these static assays of pioneer function with investigation of how protein domains contribute to protein dynamics in vivo, our data support an essential role for the N terminus in promoting the stable chromatin occupancy necessary for binding closed but not accessible chromatin. Furthermore, both domains promote localized chromatin scanning, indicated by less compact trajectories when these domains are mutated. Together, these data demonstrate that the structured DBD and the disordered N terminus are required in combination for the stable chromatin occupancy required for pioneer function. These requirements likely reflect complex engagements with chromatin that are only partially reflected in the ability to bind mononucleosomes in vitro.

Despite contributing to Grh pioneering function, the N terminus is largely unstructured and lacks sequence conservation. There is a single, structured domain whose function is unclear. While it was initially defined as the minimal transactivation domain in *Drosophila* cells, when expressed in yeast, this domain does not activate reporter gene expression (*49*). Furthermore, we showed that the minimal transactivation domain (SD) is not required for activation of a reporter gene nor is it sufficient to promote wild-type levels of activation. While these data demonstrate that the structured domain is not, as previously suggested, the only transactivation domain, addition of this domain to a scrambled N terminus increased binding and opening of closed chromatin, suggesting that it promotes pioneer activity.

Apart from the structured region, the N terminus contributes to pioneering through a mechanism that is, at least in part, independent of amino acid sequence. This activity is similar to what has been demonstrated for activation domains of yeast transcription factors, two-thirds of which retain the ability to activate gene expression when scrambled (*75*). Disordered regions are a shared feature of many eukaryotic transcription factors and contribute to chromatin binding through multiple mechanisms including directly engaging with chromatin and facilitating increased local concentrations through multivalent interactions. While the N terminus of human GRHL2 is shorter and has less predicted disorder than *Drosophila* Grh, it enables the stable chromatin occupancy required for binding and opening closed chromatin. Similarly, the *Caenorhabditis elegans* Grh ortholog, which is also truncated relative to *Drosophila* Grh, partially rescues embryonic viability and chromatin accessibility in a *Drosophila grh*-null background (*50*). These data support a role for weak, nonspecific, multivalent interactions mediated by disordered N termini in promoting pioneer activity. By contrast, the scrambled N terminus of Grh and the N terminus of GRHL2 do not allow for activation of a reporter gene, separating pioneering from transcriptional activation and suggesting that sequence-specific protein interactions may be required for activation.

Using multiple, complementary assays, we define how protein-intrinsic features contribute to the defining features of the pioneer factor Grh. Both sequence-specific interactions with DNA, driven by the conserved DBD, and nonspecific interactions, mediated by the unstructured N terminus, contribute to the stable chromatin occupancy required for Grh pioneer-factor activity. We suggest that despite having distinct polypeptide sequences, pioneer factors require stable chromatin occupancy, which can be mediated through diverse mechanisms. Many pioneer factors, including Grh, are dysregulated in cancers, and discrete protein domains can be brought together through oncogenic fusions. Thus, our analysis of how multiple protein domains contribute to pioneer activity has important implications for both normal development and cancer.

## MATERIALS AND METHODS

### Experimental design

#### Nucleosome reconstitution

The 159-bp endogenous DNA fragment (dm3_chr2R: 19,981,929-19,982,087) was amplified from *Drosophila* genomic DNA. For Widom 601 nucleosomes, the Grh motif (AACCGGTT) was inserted into the 153-bp Widom 601 sequence at SHL -6.5, −4.5, −4.0, and 0 (the dyad) (*53*). DNA was amplified using one Cy5-labeled primer and one unlabeled primer. Widom 601 without the Grh motif was used as a control and amplified using one Cy3-labeled primer and one unlabeled primer. The DNA was ethanol precipitated and purified with AxyPrep MAG PCR Clean-Up beads (Axygen) using a 1.8× ratio of beads to sample. The nucleosomes were reconstituted using salt gradient dialysis (*76*). The Grh motif is underlined, and mutated base pairs (from canonical Widom 601 sequence) are shown in lowercase: endogenous DNA, Cy5-CCATTTTGGCGGTGGATCCAAGCGGCCAAGTGCTAATGCCCCCAGCCCCGCCGATTGCTATGTCTTTGAGCTCCAACTTGTCACGCTGCCACAGACTGGAGCTCTCTCTCCGCGAAAACCGGTTCTCTGGTGGCCGGTCGCAAACGAAATCTCCATCTG; Widom 601 DNA, Cy3-ATCCTGGAGAATCCCGGTGCCGAGGCCGCTCAATTGGTCGTAGACAGCTCTAGCACCGCTTAAACGCACGTACGCGCTGTCCCCCGCGTTTTAACCGCCAAGGGGATTACTCCCTAGTCTCCAGGCACGTGTCAGATATATACATCCTGTGAT; Widom 601 DNA with Grh motif at SHL −6.5, ATCCTGaAccggttCGGTGCCGAGGCCGCTCAATTGGTCGTAGACAGCTCTAGCACCGCTTAAACGCACGTACGCGCTGTCCCCCGCGTTTTAACCGCCAAGGGGATTACTCCCTAGTCTCCAGGCACGTGTCAGATATATACATCCTGTGAT-Cy5; Widom 601 DNA with Grh motif at SHL −4.5, ATCCTGGAGAATCCCGGTGCCGAGGCCaacCggTTGGTCGTAGACAGCTCTAGCACCGCTTAAACGCACGTACGCGCTGTCCCCCGCGTTTTAACCGCCAAGGGGATTACTCCCTAGTCTCCAGGCACGTGTCAGATATATACATCCTGTGAT-Cy5; Widom 601 with Grh motif at SHL −4.0, ATCCTGGAGAATCCCGGTGCCGAGGCCGCTCAAccGGtTGTAGACAGCTCTAGCACCGCTTAAACGCACGTACGCGCTGTCCCCCGCGTTTTAACCGCCAAGGGGATTACTCCCTAGTCTCCAGGCACGTGTCAGATATATACATCCTGTGAT-Cy5; and Widom 601 with Grh motif at SHL0, ATCCTGGAGAATCCCGGTGCCGAGGCCGCTCAATTGGTCGTAGACAGCTCTAGCACCGCTTAAACGCACGTAacCGgTtTCCCCCGCGTTTTAACCGCCAAGGGGATTACTCCCTAGTCTCCAGGCACGTGTCAGATATATACATCCTGTGAT-Cy5.

#### Protein expression and purification

As described previously, maltose-binding protein (MBP)-tagged Grh^ΔN^ (Grh^603–1032^) was purified from *Escherichia coli* (*77*, *78*) with the exclusion of the final dialysis step. Briefly, protein was bound to amylose resin (New England Biolabs) and eluted with 20 mM maltose. Baculovirus expression was used to purify full-length Grh-PH as described previously (*77*). For purification of Grh^C800A,K807A^ and Grh^R806A^, cDNA encoding each mutant was cloned into pDEST8 and used to generate baculovirus using the Bac-to-Bac expression system (Thermo Fisher Scientific). Freshly amplified virus was used to infect Sf9 cells grown in cytiva SFX media (Thermo Fisher Scientific) supplemented with 10% fetal bovine serum (FBS). As in (*13*), 3 days following infection, cells were collected on ice, centrifuged, washed with phosphate-buffered saline (PBS) containing 5 mM MgCl_2_, and centrifuged once more. The cells were resuspended in hypotonic buffer [15 mM Hepes, 15 mM KCl, 2 mM MgCl_2_, 0.02% Tween, 10% glycerol, 2 mM β-mercaptoethanol, 1 mM EGTA, 0.4 mM phenylmethylsulfonyl fluoride (PMSF), and Pierce protease inhibitor tablets (Thermo Fisher Scientific)], flash frozen in liquid nitrogen, and kept at −80°C. Cell suspensions were thawed and dounced. KCl was then added to bring the concentration to 300 mM, and the lysate was cleared by centrifugation (10,000 rpm for 10 min at 4°C, 16,000 rpm for 10 min at 4°C). PMSF (20 μM) and 750 μl of prewashed anti-flag M2 affinity beads (Sigma-Aldrich A2220) were added to the extract and incubated at 4°C for 3 hours. The beads were washed, and protein was eluted in buffer containing 150 mM KCl and Flag peptide (200 μg/ml) following a 30-min incubation with end-over end mixing at 4°C. Protein concentration was determined by comparing levels to bovine serum albumin (BSA) standards using Coomassie blue staining.

#### Electrophoretic mobility shift assays

Cy5-labeled probe [2.5 nM (50 fmol/20 μl reaction); DNA or nucleosome] was incubated with recombinant Grh protein in buffer containing 5 ng of poly[d(I-C)], 12.5 mM Hepes, 0.5 mM EDTA, 0.5 mM EGTA, 5% glycerol, 0.25 mM dithiothreitol, 150 μM PMSF, BSA (0.075 mg/ml), 2.5 mM MgCl_2_, 0.005% Tween, and 50 mM KCl at room temperature for 60 min (30 min for Fig. 1A). For reactions containing both Cy5- and Cy3-labeled Widom 601 DNA or nucleosomes, each was present at 2.5 nM (5 nM total). Reactions were run on a 4% nondenaturing polyacrylamide gel in 0.5× tris-borate EDTA. Gels were visualized with a Typhoon FLA9000 using Cy5 and Cy3 fluorescence settings. The fraction of bound protein was quantified by dividing the shifted band of each respective reaction by the unshifted band from the no protein reaction. The relative fraction shifted was quantified by subtracting the fraction of bound protein with the motif-containing Cy5 nucleosomes from the fraction of bound protein with the Cy3 nucleosomes lacking the motif. Quantification was performed on three or four independent gels.

#### Cell culture and generation of stable cell lines

We used a stable cell line expressing full-length wild-type Grh that was generated previously (*14*). There is an alternative ATG 252 bp after the initial start codon, resulting in multiple protein products upon copper induction. Additional expression constructs were generated as described below and cloned into pMT-puro. Point mutants, Grh^C800A,K807A^ and Grh^R806A^, were generated with site directed mutagenesis. Grh^Scr^ was made by randomizing the amino acids between 2 and 602 in Grh using the Peptide Nexus server (*79*). Codon composition was retained, and the C-terminal amino acids 603 to 1032 were not modified. The structured minimal activation domain (Grh^173–228^) (*49*) was added back to Grh^Scr^, replacing amino acids 173 to 228 with the wild-type sequence to generate Grh^Scr + SD^. An N-terminal HA tag was added to the cDNA encoding human GRHL2 (isoform 1). As a control, an N-terminal HA tag was added to *Drosophila* Grh. Chimeric constructs were designed based on conserved regions defined by Traylor-Knowles *et al.* (*35*): HA-GRHL2.Grh (GRHL2^1–246^ fused to Grh^633–1032^) and HA-Grh.GRHL2 (Grh^1–632^ fused to GRHL2^247–624^). Stable cell lines were generated as described previously (*14*). S2 cells were cultured at 26°C in Schneider’s medium (Thermo Fisher Scientific) supplemented with 10% FBS (Omega Scientific) and antibiotic-antimycotic (Thermo Fisher Scientific). Cells were plated at 0.5 × 10^6^ cells per ml. After 24 hours, cells were transfected with 10 μg of plasmid DNA using Effectene Transfection Reagent (QIAGEN). After an additional 24 hours, puromycin (Thermo Fisher Scientific) was at a final concentration of 2 μg/ml. Cells were recovered after 2 to 3 weeks of selection. Following recovery of stable cell lines, cells were cultured with 1 μg/ml of puromycin.

#### Induction of TF expression

Cells were plated at 1 × 10^6^ cells per ml, and protein expression was induced by adding copper sulfate at the following concentrations: 100 μM Grh, 100 μM Grh^C800A,K807A^, 100 μM Grh^R806A^, 400 μM Grh^Scr^, 100 μM Grh^Scr + SD^, 200 μM HA-Grh, 450 μM HA-GRHL2, 450 μM HA-GRHL2.Grh, and 450 μM HAGrh.GRHL2. Concentration of copper sulfate used for induction was determined based on approximation to physiological levels of expression as determined empirically by immunoblots (*14*). Cells were collected 48 hours following induction for immunoblotting, ChIP-seq, or ATAC-seq.

#### Luciferase assays

The promoter of *hgo*, including the upstream Grh-binding site, was cloned into pGL3-Basic to drive Firefly luciferase expression (*68*). cDNAs were cloned into pAc5.1 for Grh protein expression. As previously published, transient transfections were performed in triplicate with 90 ng of wild-type or mutant *hgo* reporter plasmid, 100 ng of Grh-expression plasmid, 10 ng of actin-Renilla plasmid, and 100 ng of empty expression plasmid using the Effectene Transfection Reagent (QIAGEN) (*68*). Luciferase assays were performed on cell lysate using the Dual-Luciferase Assay Kit (Promega). Fold activation was calculated relative to luciferase reads from controls transfected with 200 ng of empty expression plasmid in place of the Grh-expression plasmid.

#### Immunoblotting

Proteins were separated by SDS–polyacrylamide gel electrophoresis and transferred to a 0.45-μm polyvinylidene difluoride (PVDF) membrane at 4°C in transfer buffer (20% methanol, 25 mM tris, and 200 mM glycine) for 60 min at 500 mA. Membranes were blocked at room temperature in BLOTTO (2.5% nonfat dry milk, 0.5% BSA, and 0.5% NP-40 in TBST) for 30 min at room temperature. Primary antibody incubations were performed overnight at 4°C at the following concentrations: anti-Grh (1:1000) (*77*), anti-HA-peroxidase (1:500) (clone 3F10, Roche), and anti-tubulin (1:10,000) (DM1A, Sigma-Aldrich). Secondary antibody incubation with goat anti-rabbit immunoglobulin G (IgG)–horseradish peroxidase (HRP) (1:5000) (Bio-Rad) or goat anti-mouse IgG-HRP (1:6000) (Bio-Rad) was for 1 hour at room temperature. HRP activity was detected using SuperSignal West Pico PLUS chemiluminescent substrate (Thermo Fisher Scientific). Imaging was performed using film or an Azure Biosystems c600 imaging system.

#### ChIP sequencing

ChIP-seq was performed as described previously (*14*). Briefly, two replicates were collected with 25 × 10^6^ cells per replicate. Samples were fixed in 0.8% formaldehyde for 7 min and quenched with 125 mM glycine. Fixed chromatin was sonicated on a Covaris S220. Immunoprecipitations (IPs) were incubated with 8 μl of anti-Grh (*77*) or 7.5 μl of anti-HA (12CA5, Sigma-Aldrich) at 4°C overnight and purified using Dynabeads Protein A for Grh (20 μl; Thermo Fisher Scientific) or M-280 Sheep Anti-Mouse IgG for HA (60 μl; Thermo Fisher Scientific). The beads were washed, and chromatin was eluted. IPs and inputs were treated with ribonuclease A, and cross-links were reversed. DNA was isolated by phenol:chloroform extraction and concentrated overnight by ethanol precipitation. Libraries were prepared using the NEB Next Ultra II Kit. Grh-mutant libraries (Fig. 2) were sent to the Northwestern Sequencing Core (NUSeq) for sequence on the Illumina NextSeq 500 using 75-bp single-end reads; additional reads were sequenced on the Illumina HiSeq 4000 using 50-bp single-end reads. Grh^Scr^ libraries (Fig. 4) were sent to NUSeq for sequencing on the Illumina NovaSeq X Plus using 50-bp paired-end reads. GRHL2 libraries (Fig. 5) were sent for sequencing at the UW Madison Biotechnology Center on the Illumina NovaSeq X Plus using 150-bp paired-end reads.

#### ATAC sequencing

ATAC-seq was performed as described previously (*14*). For each experiment, two replicates were collected with 2 × 10^5^ cells per replicate. Cells were lysed in ATAC lysis buffer (10 mM tris, 10 mM NaCl, 3 mM MgCl_2_, and 0.1% NP-40), resuspended in buffer TD (Illumina), and tagmented with Tn5 enzyme (Illumina). DNA was purified with the MinElute Reaction Cleanup Kit (QIAGEN), and polymerase chain reaction (PCR)–amplified with IDT for Illumina Nextera DNA Unique Dual Indices in NEBNext Hi-Fi 2× PCR Master Mix. Libraries were purified with Axygen paramagentic beads (Thermo Fisher Scientific) at a ratio of 1.2× and eluted in 10 mM tris. Samples were sequenced at the UW Madison Biotechnology Center on the Illumina NovaSeq X Plus using 150-bp paired-end reads.

#### Imaging mitotic retention

cDNAs encoding Grh, Grh^ΔN^, Grh^R806A^, or GRHL2 were cloned into pAc5.1 with an N-terminal GFP tag. Cells were plated in 35-mm glass bottom dishes at 1.0 × 10^6^ cells/ml and incubated at 25°C for 30 min to allow for adherence. Cells were then transfected with 250 ng of plasmid encoding GFP-Grh and 250 ng of pAc-mCh-tubulin (DGRC stock no. 1462) (*80*) using the Effectene Transfection Reagent (QIAGEN). For fig. S6A, cells were treated with Hoechst 33342 to label the DNA. Cells were imaged 48 hours after transfection on a Nikon Eclipse Ti2-E inverted fluorescence microscope. Mitotic cells were visually identified using mCh-tubulin to identify the spindle and then imaged through mitosis with a 60× objective every 10 s.

#### Drosophila *culture and transgene expression*

All flies were grown on molasses at 25°C unless otherwise noted. Transgenic flies were made by cloning cDNAs encoding Grh, Grh^ΔN^, Grh^R806A^, or GRHL2 into pUASt-attB with an N-terminal Halo tag and integrated at docking site VK14 on chromosome II using ΦC31-mediated integration (BestGene, Chino Hills, CA). Transgenic expression was driven in a temperature-dependent manner. Fly lines carrying transgenes for Grh expression were crossed to *MS1096-GAL4,tubulin-GAL80^ts^* (*81*) and raised at 18°C. Larvae were moved to 30°C for 24 hours prior to dissection.

#### Salivary gland dissections and Halo ligand treatment

Salivary glands were dissected from third-instar larvae of the selected genotype in Schneider media supplemented with 5% FBS and 1× antibiotic-antimycotic. Glands were incubated for 15 min with Halo ligands: 200 nM JF646 (Promega) for FRAP and imaging or 0.5 nM TMR (Promega) for single-molecule tracking. Following incubation, glands were washed three times for 10 min each in media followed by a PBS rinse. Glands were then mounted onto polylysine-coated coverslips in Schneider media with 2.5% (w/v) methyl-cellulose as described previously (*82*).

#### Fluorescence recovery after photobleaching

Imaging of the salivary glands was performed on JF646-labeled salivary glands as described by DeHaro-Arbona *et al*. (*81*) Individual nuclei were imaged with a 4.5× zoom, 512 by 512 pixel resolution, and settings were optimized for bleaching and scanning speed: Pinhole was opened to 3.5-Airy; speed was 700 Hz. Effective bleaching was achieved by point bleaching. Images before and after bleaching were acquired every 0.4 s. After 50 images postbleaching, frame gap was increased to 1 s to minimize unintentional bleaching. FRAP curves were normalized as described by DeHaro-Arbona *et al.* (*81*) His2Av-iRFP was imaged as a control.

#### Single-molecule tracking

Single-molecule imaging was performed using a custom-built microscope as described previously (*82*, *83*). The TMR Halo ligand was excited at 561 nm under continuous illumination, and emission was collected at 585 nm. Between five and eight nuclei (Grh, 8; Grh^ΔN^, 6; Grh^R806A^, 5) were imaged with a 50-ms exposure time for 5 min.

### Statistical analysis

#### ChIP sequencing analysis

Data were analyzed as previously described (*14*). Briefly, Reads were aligned to the *D. melanogaster* genome (dm6) using Bowtie2 (v2.4.4). Unmapped reads, reads aligning to the mitochondrial genome or unplaced scaffolds, reads aligning more than once, or low-quality reads (MAPQ < 30) were removed. Peaks were called with MACS2 (v2.2) with the corresponding input as control; only peaks detected in both replicates were retained for downstream analyses. After peak calling, peaks were filtered for a MACS2 *q* value greater than 10 and a MACS2 enrichment score greater than 3. These parameters were similarly used to filter published classes defined by Gibson *et al.* (*14*). To generate bigWigs, we used deeptools bamCoverage (v3.5.1) and *z*-score normalized across the genome.

#### ATAC sequencing analysis

Reads were processed as previously described (*14*). In brief, reads were trimmed for adapter sequences using NGmerge and aligned to the *D. melanogaster* genome (dm6) using Bowtie2 (v2.4.4). Unmapped reads, reads aligning to the mitochondrial genome or unplaced scaffolds, reads aligning more than once, or low-quality reads (MAPQ < 30) were removed. Only fragments shorter than 100 bp were used for downstream analysis. Peaks were called with MACS2 (v2.2), and only peaks detected in both replicates were retained for downstream analysis. bigWig files were generated with deepTools bamCoverage (v3.5.1) and *z*-score normalized across the genome. Differential accessibility was determined using DESeq2 and compared induced samples to uninduced controls.

#### Single-molecule tracking analysis

Analysis of single molecule trajectories was performed using a previously described pipeline (*83*). Briefly, single molecules were localized using a Gaussian fitting–based approach (*84*) and tracked using a multiple hypothesis tracking algorithm (*85*). Trajectories with a minimum of four time points were analyzed using a vbSPT (*70*) and segregated into one of three states (D1, D2, or D3) defined by a unique Brownian motion diffusion coefficient.

#### Angle and anisotropy analyses

Anisotropy analyses were done as previously described except that only D3 (as identified by vbSPT) was used for analysis, as molecular displacement for this population exceeded the localization error, and hence, angles could be accurately calculated (*83*, *86*). The fold anisotropy metric *f*(180/0) was calculated as follows

$$f\left(180/0\right)=\text{fold}\;\left(\frac{180{}^{\circ}\pm 30{}^{\circ}}{0{}^{\circ}\pm 30{}^{\circ}}\right)$$

## Supplementary Materials

### This PDF file includes:

Figs. S1 to S8 References
