# Supplementary material for: Pioneer-factor activity requires stable chromatin occupancy mediated by both sequence-specific binding and disordered protein domains
Source: Sci Adv. 2026 Jun 19;12(25):eaef2244. doi: 10.1126/sciadv.aef2244 (PMC13281792; doi:10.1126/sciadv.aef2244)
Supplement: Supplementary file 1 — Figs. S1 to S8 References [file sciadv.aef2244_sm.pdf]

Science Advances
AAAS

Supplementary Materials for

Pioneer-factor activity requires stable chromatin occupancy mediated by both sequence-specific binding and disordered protein domains

Meghan M. Freund *et al.*

Corresponding author: Melissa M. Harrison, mharrison3@wisc.edu

Sci. Adv. **12**, eaef2244 (2026)
DOI: 10.1126/sciadv.aef2244

This PDF file includes:

Figs. S1 to S8
References

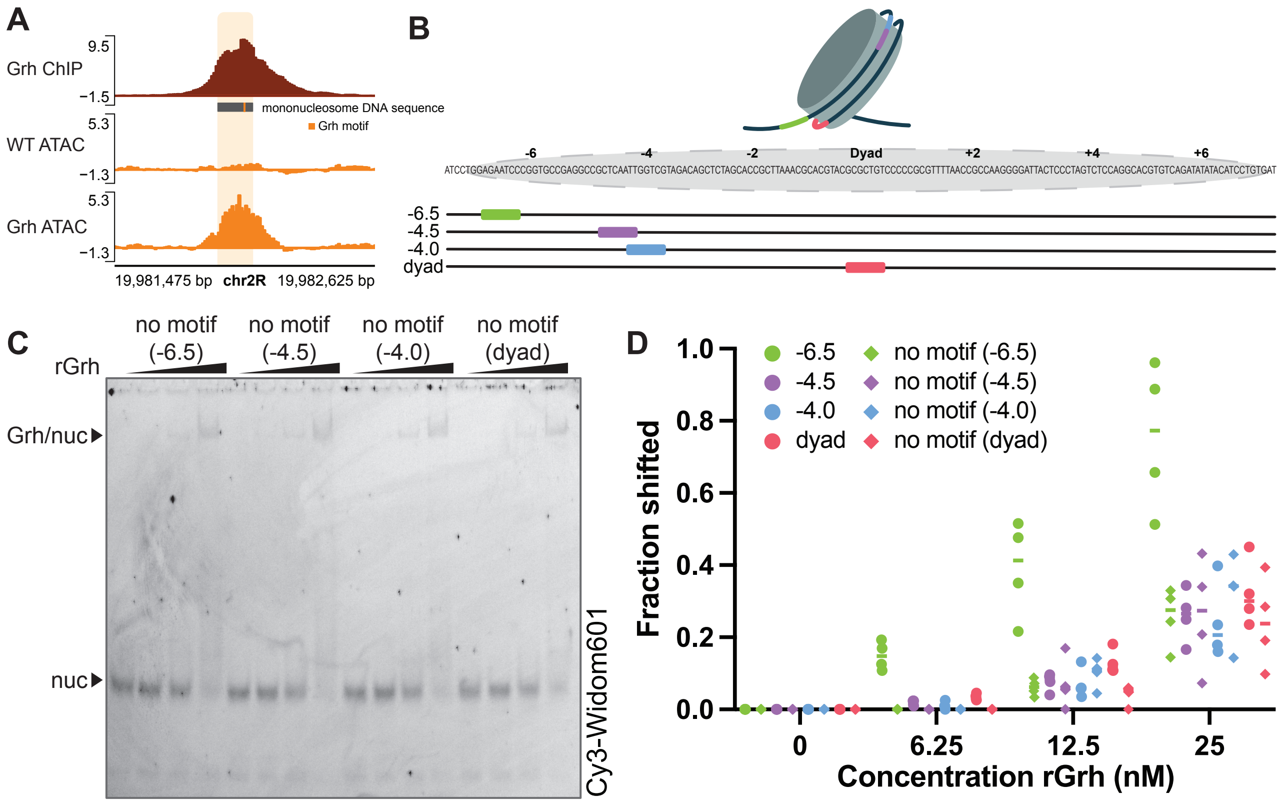

Figure S1. Nucleosomal DNA sequences (A) Genome browser tracks of the endogenous region used to make mononucleosomes. ATAC-seq data before induction (WT ATAC) and upon expression of Grh (Grh ATAC) demonstrate the increase in chromatin accessibility upon Grh expression. Grh ChIP-seq data upon expression of Grh (Grh ChIP). Sequence used for mononucleosome DNA is marked in grey with the Grh motif highlighted in orange. (B) Widom 601 sequence with Grh motifs marked at the noted positions. (C) EMSA with increasing concentrations of recombinant Grh (rGrh) (0 - 25nM) incubated with Cy3-labeled Widom 601 nucleosomes without the Grh motif. Same gel as Figure 1B but imaged for Cy3. (D) Quantification of the fraction of Grh-bound nucleosomes, indicated by the proportion shifted in the EMSA shown in Figure 1B, S1C and three additional experiments.

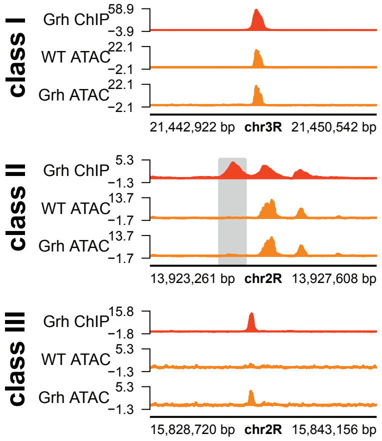

Figure S2. Grh binds and opens chromatin in S2 cells. Genome browser tracks showing ChIP-seq signal in cells expressing ectopic Grh and ATAC-seq signal before (WT ATAC) and after (Grh ATAC) induction of Grh. Example track shown of all three classes. Class I: accessible and bound by Grh. Class II: inaccessible and bound by Grh. Class III: inaccessible, bound and opened by Grh (as shown in Gibson et al. 2024 (*14*)).

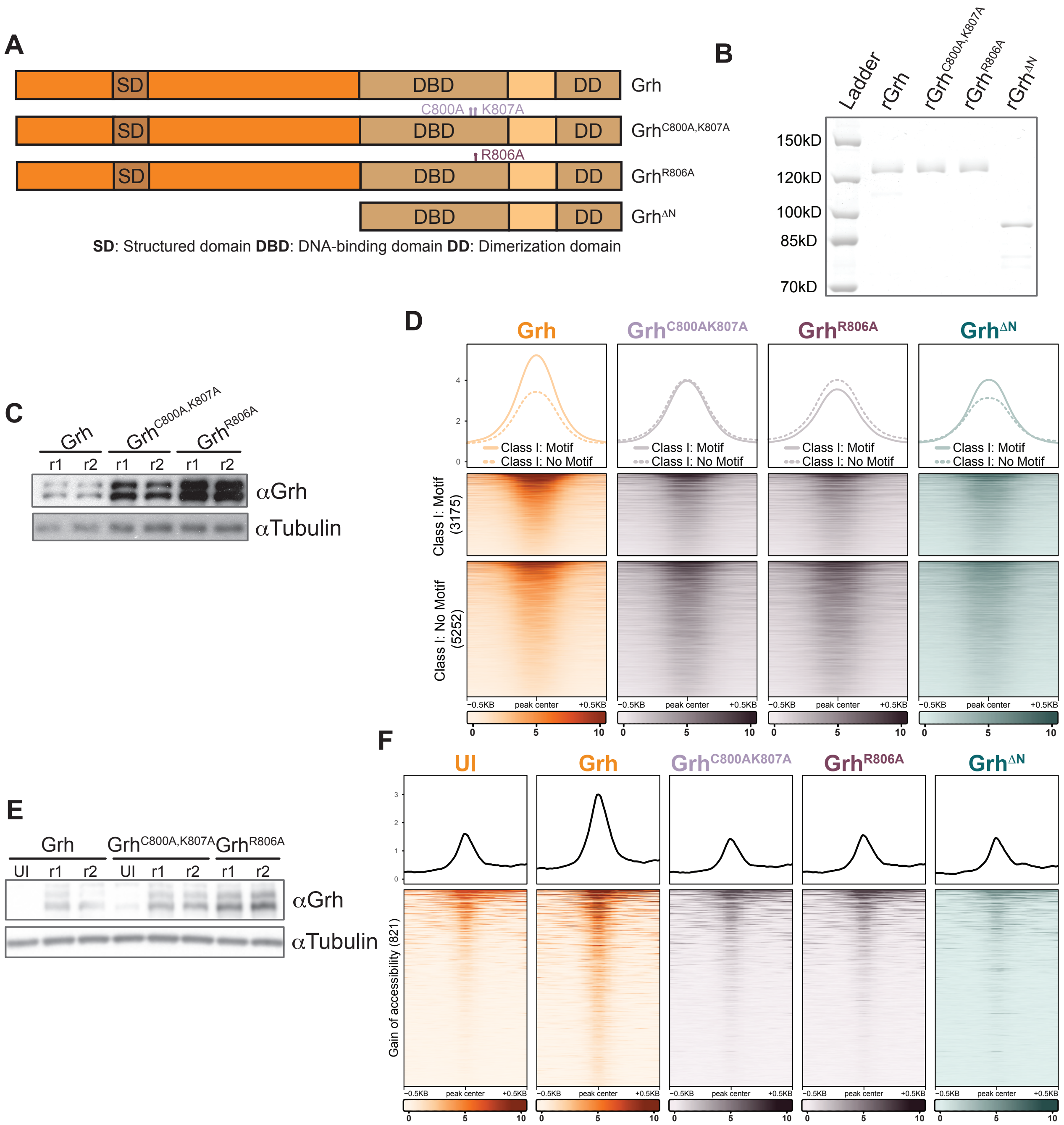

Figure S3. Grh requires the DBD and regions outside for binding and opening chromatin. (A) Models of Grh proteins and mutants with domains indicated. (B) Coomassie stain of an SDS-PAGE gel of recombinant Grh proteins: wild-type Grh, Grh^{C800A,K807A}, Grh^{R806A} and Grh$^{\Delta N}$. (C) Western blot showing expression of wild-type Grh, Grh^{C800A,K807A} and Grh^{R806A} in S2 cells used for ChIP-seq in Figure 2E. (D) Heatmaps and metaplots showing Grh binding (ChIP-seq) at Class I sites containing the canonical Grh motif or without the motif (no motif). (E) Western blot showing expression of wild-type Grh, Grh^{C800A,K807A} and Grh^{R806A} in S2 cells used for ATAC-seq in Figure 2D. UI, uninduced, r1; replicate one, r2; replicate two. (F) Heatmaps and metaplots for ATAC-seq of regions that gain accessibility upon expression of any one of the proteins indicated above. UI, uninduced.

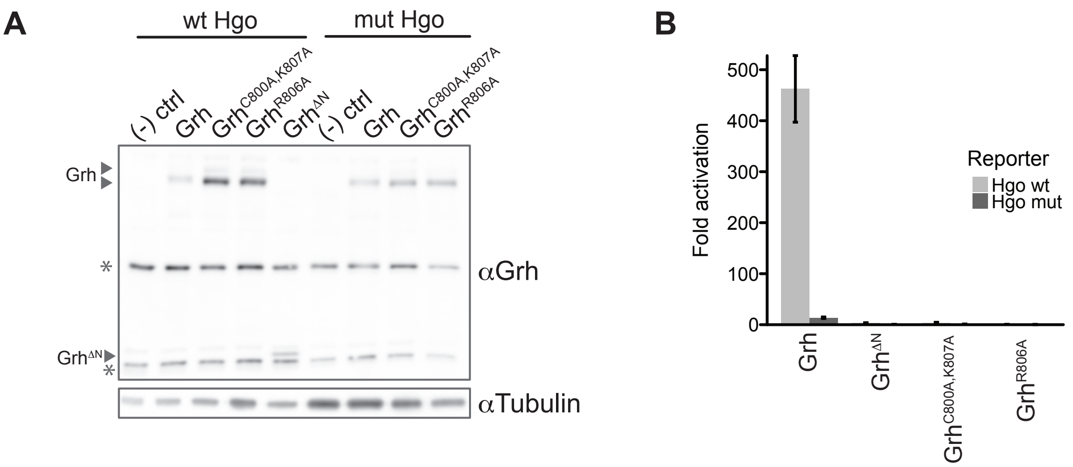

Figure S4. Grh requires the DBD and regions outside for activating gene expression in cell culture. (A) Western blot showing expression levels of wild-type Grh, Grh^{C800A,K807A}, Grh^{R806A} and Grh$^{\Delta N}$ in S2 cells used for luciferase assay in G. *, nonspecific background (B) Fold activation of the wild-type or mutant *hgo* luciferase reporter by either wild-type Grh, Grh^{C800A,K807A}, Grh^{R806A} or Grh$^{\Delta N}$. n = 3, mean ± S.D.

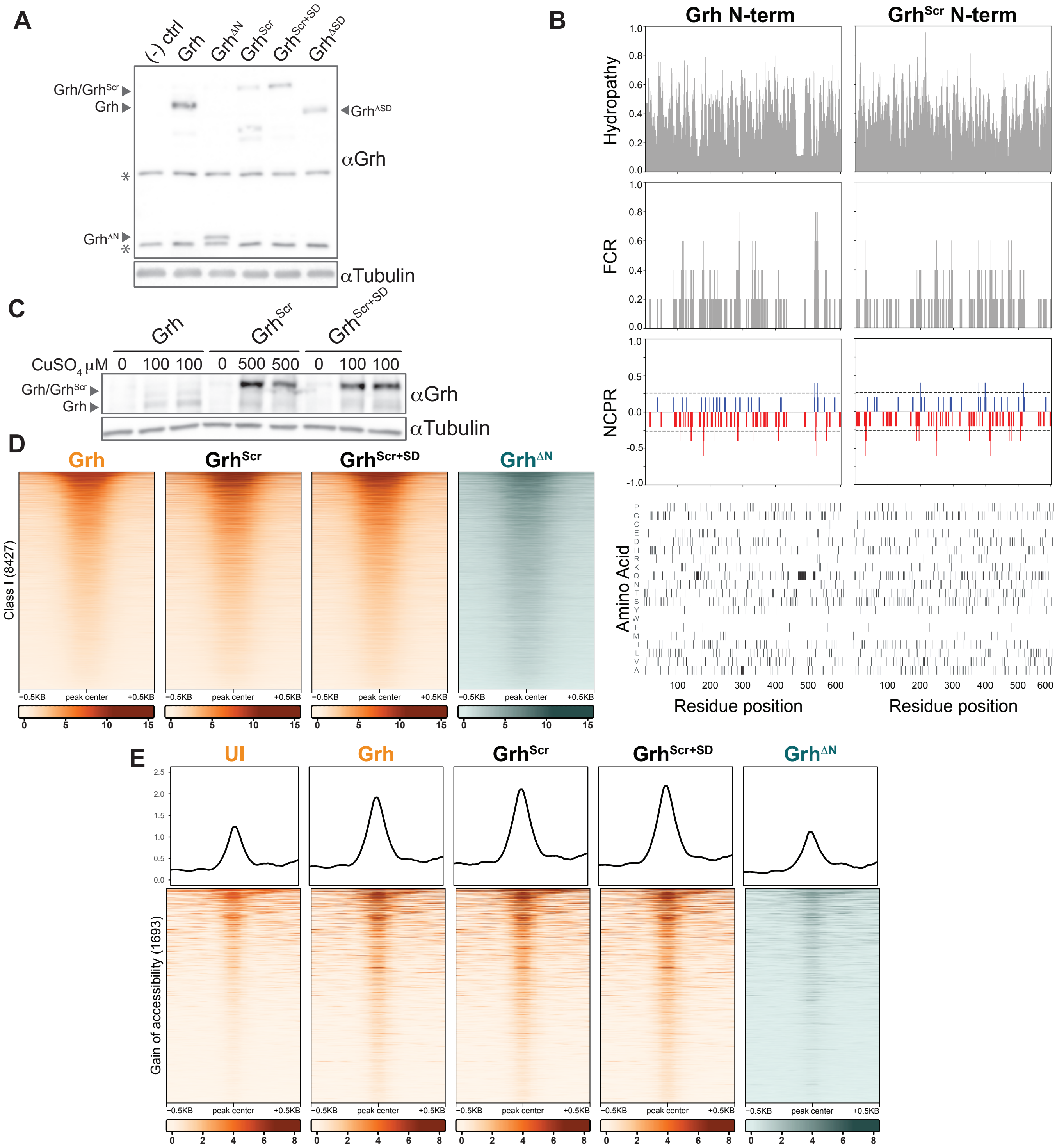

Figure S5. The intrinsically disordered N-terminus can contribute to pioneering independed of amino acid sequence. (A) Western blot showing expression levels of wild-type Grh, $Grh^{\Delta N}$, Grh^{Scr}, Grh^{Scr+SD} and $Grh^{\Delta SD}$ in S2 cells used for luciferase assay in Figure 4B. *, nonspecific background (B) Mean hydropathy, fraction of charged residues (FCR) and net charge per residue (NCPR) was calculated for groups of 5 residues (blob 5) with CIDER (88). Amino acid composition is shown below. (C) Western blot showing expression of wild-type Grh, Grh^{Scr} and Grh^{Scr+SD} in S2 cells used for ChIP-seq and ATAC-seq in Figure 4C and 4D. (D) Heatmaps of Grh binding (ChIP-seq) for the proteins indicated above at 8247 Class I sites (defined in Gibson et al. 2024 (*14*)). (E) Heatmaps and metaplots for ATAC-seq of regions that gain accessibility upon expression of any one of the proteins indicated above. UI, uninduced.

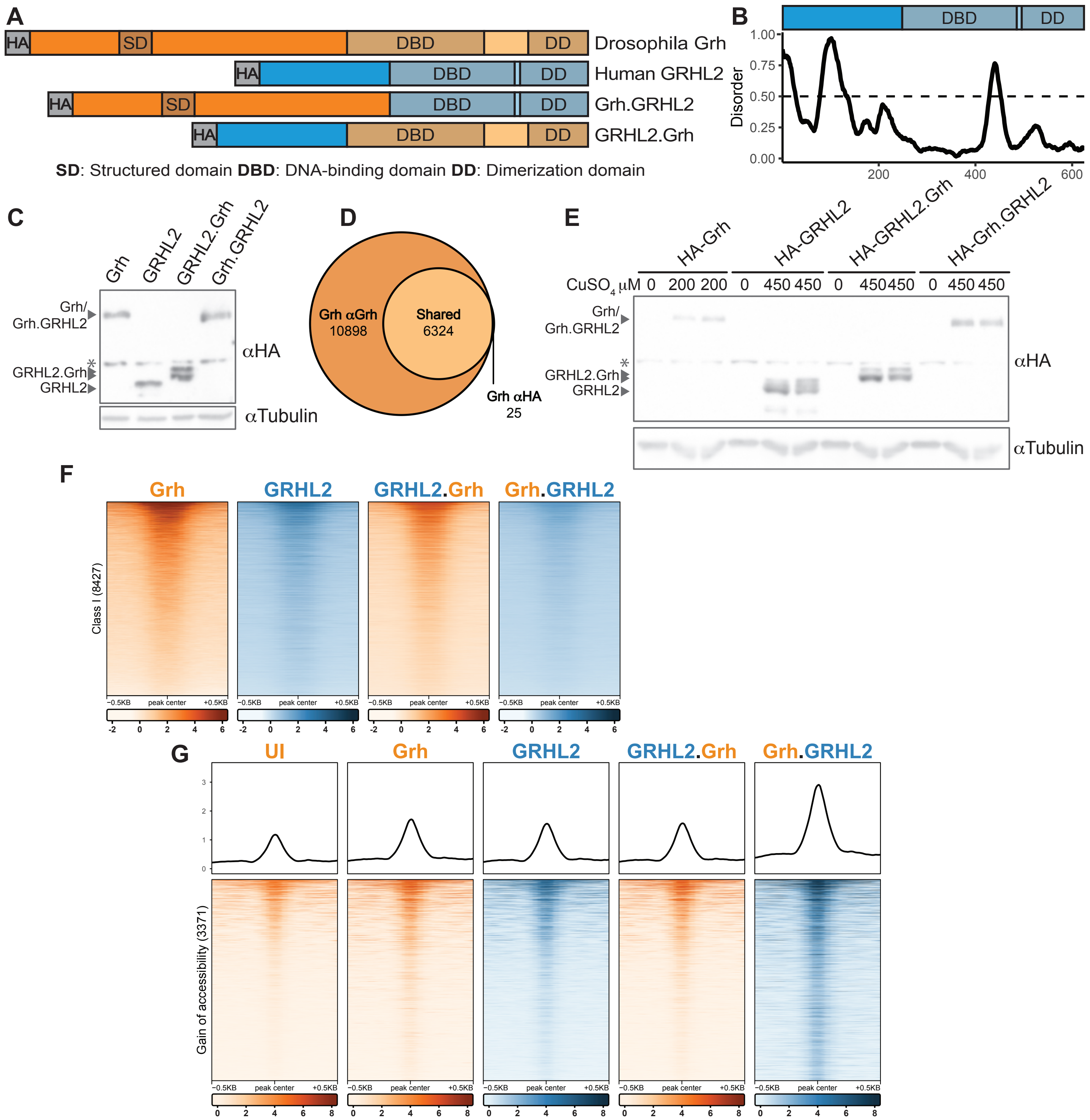

Figure S6. Diverse N-terminal domains are sufficient for pioneering. (A) Models of Grh proteins and chimeras expressed with domains indicated. Blue indicates amino acids from humans and orange indicates amino acides from *Drosophila*. (B) Graph of predicted disorder score (from Metapredict (87)) for GRHL2. (C). Western blot showing expression levels of *Drosophila* Grh, human GRHL2, or chimeras (GRHL2.Grh, Grh.GRHL2) in S2 cells used for luciferase assay in Figure 5A. *, nonspecific background (D) Overlap between peaks called using the αGrh antibody or αHA antibody. (E) Western blot showing expression of *Drosophila* Grh, human GRHL2, or chimeras (GRHL2.Grh, Grh.GRHL2) in S2 cells used for ChIP-seq and ATAC-seq in Figure 5B and C. *, nonspecific background (F) Heatmaps of Grh binding (ChIP-seq) for the proteins indicated above at 8247 Class I sites (defined in Gibson et al. 2024 (*14*)). (G) Heatmaps and metaplots for ATAC-seq of regions that gain accessibility upon expression of any one of the proteins indicated above. UI, uninduced.

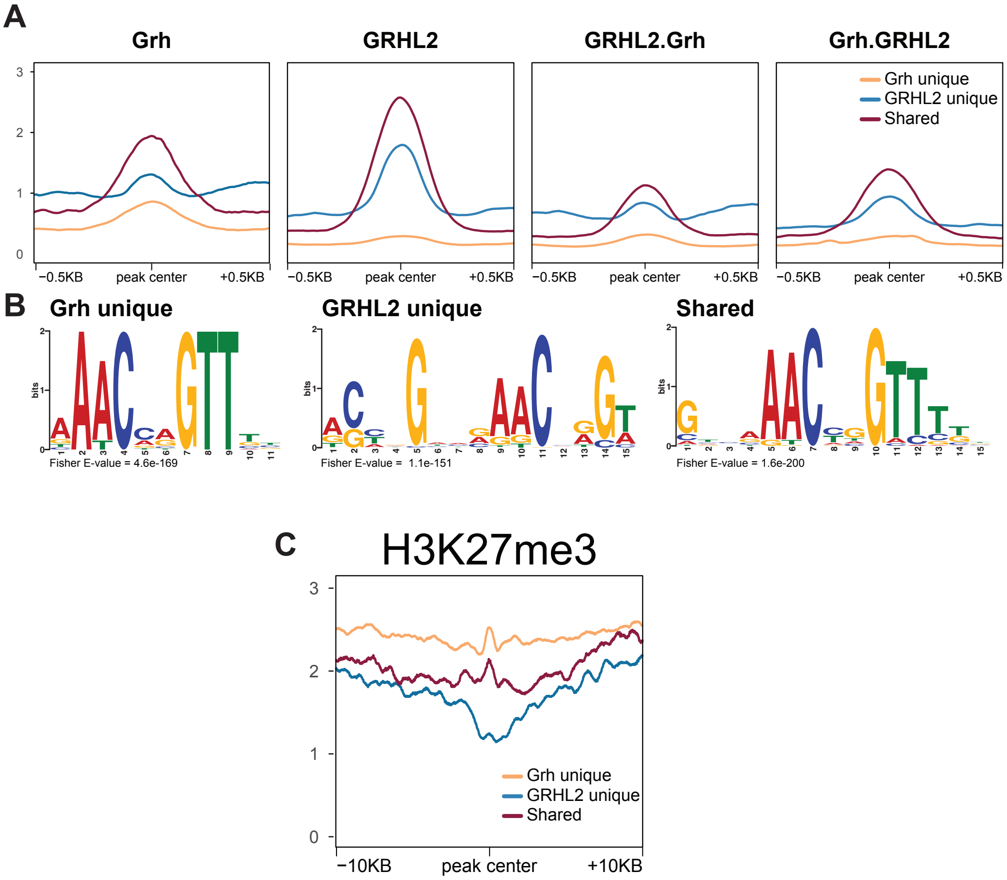

Figure S7. GRHL2 binds Grh motifs in closed chromatin. (A) Metaplots showing enrichment of binding at inaccessible sites unique to Grh or GRHL2 and shared sites. (B) Top motif enrichment for inaccessible sites uniquely bound by Grh or GRHL2 and shared sites identified by MEME-suite (89). (C) Metaplot of H3K27me3 levels surrounding inaccessible sites unique to Grh or GRHL2 and shared sites.

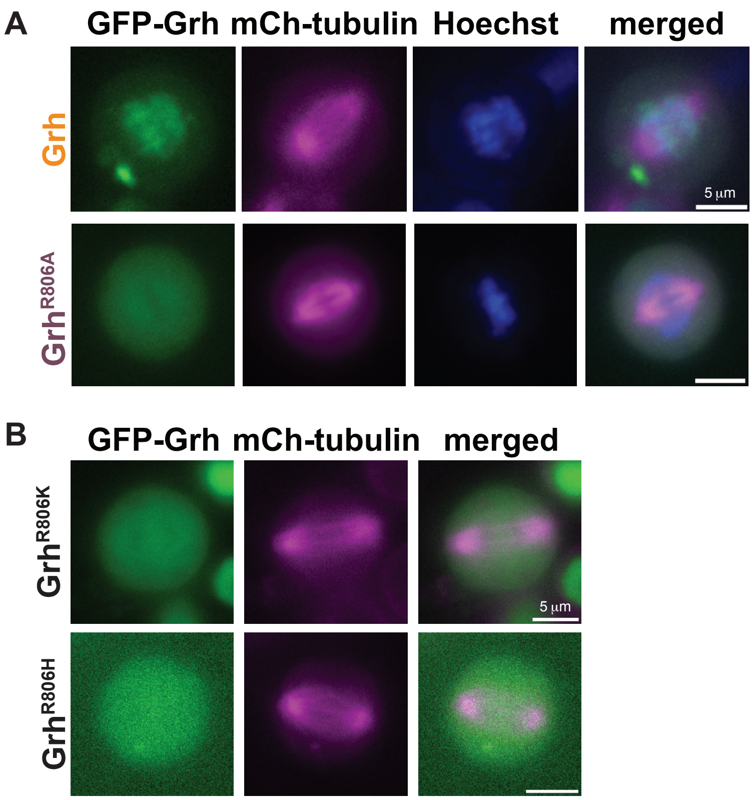

Figure S8. Disruption of charge is not resposible for loss of Grh retention. (A) Images of GFP-tagged Grh proteins (green), mCherry-tubulin (magenta), and DNA labelled with Hoechst (blue) at anaphase in S2 cells. (B) As in (A) except DNA was not labelled.

REFERENCES

1. X.-Y. Li, S. Thomas, P. J. Sabo, M. B. Eisen, J. A. Stamatoyannopoulos, M. D. Biggin, The role of chromatin accessibility in directing the widespread, overlapping patterns of *Drosophila* transcription factor binding. *Genome Biol.* **12**, R34 (2011).

2. M. Iwafuchi-Doi, K. S. Zaret, Pioneer transcription factors in cell reprogramming. *Genes Dev.* **28**, 2679–2692 (2014).

3. A. Soufi, M. F. Garcia, A. Jaroszewicz, N. Osman, M. Pellegrini, K. S. Zaret, Pioneer transcription factors target partial DNA motifs on nucleosomes to initiate reprogramming. *Cell* **161**, 555–568 (2015).

4. K. S. Zaret, Pioneer transcription factors initiating gene network changes. *Annu. Rev. Genet.* **54**, 367–385 (2020).

5. E. D. Larson, A. J. Marsh, M. M. Harrison, Pioneering the developmental frontier. *Mol. Cell* **81**, 1640–1650 (2021).

6. S. Stoeber, H. Godin, C. Xu, L. Bai, Pioneer factors: Nature or nurture? *Crit. Rev. Biochem. Mol. Biol.* **59**, 139–153 (2024).

7. M. Bellec, J. Dufourt, G. Hunt, H. Lenden-Hasse, A. Trullo, A. Zine El Aabidine, M. Lamarque, M. M. Gaskill, H. Faure-Gautron, M. Mannervik, M. M. Harrison, J.-C. Andrau, C. Favard, O. Radulescu, M. Lagha, The control of transcriptional memory by stable mitotic bookmarking. *Nat. Commun.* **13**, 1176 (2022).

8. J. M. Caravaca, G. Donahue, J. S. Becker, X. He, C. Vinson, K. S. Zaret, Bookmarking by specific and nonspecific binding of FoxA1 pioneer factor to mitotic chromosomes. *Genes Dev.* **27**, 251–260 (2013).

9. L. A. Cirillo, Binding of the winged-helix transcription factor HNF3 to a linker histone site on the nucleosome. *EMBO J.* **17**, 244–254 (1998).

10. B. T. Donovan, H. Chen, P. Eek, Z. Meng, C. Jipa, S. Tan, L. Bai, M. G. Poirier, Basic helix-loop-helix pioneer factors interact with the histone octamer to invade nucleosomes and generate nucleosome-depleted regions. *Mol. Cell* **83**, 1251–1263.e6 (2023).

11. M. Fernandez Garcia, C. D. Moore, K. N. Schulz, O. Alberto, G. Donague, M. M. Harrison, H. Zhu, K. S. Zaret, Structural features of transcription factors associating with nucleosome binding. *Mol. Cell* **75**, 921–932.e6 (2019).

12. J. Gassler, W. Kobayashi, I. Gáspár, S. Ruangroengkulrith, A. Mohanan, L. Gómez Hernández, P. Kravchenko, M. Kümmecke, A. Lalic, N. Rifel, R. J. Ashburn, M. Zaczek, A. Vallot, L. Cuenca Rico, S. Ladstätter, K. Tachibana, Zygotic genome activation by the totipotency pioneer factor Nr5a2. *Science* **378**, 1305–1315 (2022).

13. S. L. McDaniel, T. J. Gibson, K. N. Schulz, M. Fernandez Garcia, M. Nevil, S. U. Jain, P. W. Lewis, K. S. Zaret, M. M. Harrison, Continued activity of the pioneer factor Zelda is required to drive zygotic genome activation. *Mol. Cell* **74**, 185–195.e4 (2019).

14. T. J. Gibson, E. D. Larson, M. M. Harrison, Protein-intrinsic properties and context-dependent effects regulate pioneer factor binding and function. *Nat. Struct. Mol. Biol.* **31**, 548–558 (2024).

15. J. Lerner, A. Katznelson, J. Zhang, K. S. Zaret, Different chromatin-scanning modes lead to targeting of compacted chromatin by pioneer factors FOXA1 and SOX2. *Cell Rep.* **42**, 112748 (2023).

16. S. Brodsky, T. Jana, K. Mittelman, M. Chapal, D. K. Kumar, M. Carmi, N. Barkai, Intrinsically disordered regions direct transcription factor in vivo binding specificity. *Mol. Cell* **79**, 459–471.e4 (2020).

17. D. A. Garcia, T. A. Johnson, D. M. Presman, G. Fettweis, K. Wagh, L. Rinaldi, D. A. Stavreva, V. Paakinaho, R. A. M. Jensen, S. Mandrup, A. Upadhyaya, G. L. Hager, An intrinsically disordered region-mediated confinement state contributes to the dynamics and function of transcription factors. *Mol. Cell* **81**, 1484–1498.e6 (2021).

18. F. Jonas, Y. Navon, N. Barkai, Intrinsically disordered regions as facilitators of the transcription factor target search. *Nat. Rev. Genet.* **26**, 424–435 (2025).

19. X. A. Feng, M. Yamadi, Y. Fu, K. M. Ness, C. Liu, I. Ahmed, G. D. Bowman, M. E. Johnson, T. Ha, C. Wu, GAGA zinc finger transcription factor searches chromatin by 1D–3D facilitated diffusion. *Nat. Struct. Mol. Biol.* **32**, 2359–2370 (2025).

20. X. Tang, T. Li, S. Liu, J. Wisniewski, Q. Zheng, Y. Rong, L. D. Lavis, C. Wu, Kinetic principles underlying pioneer function of GAGA transcription factor in live cells. *Nat. Struct. Mol. Biol.* **29**, 665–676 (2022).

21. S. Sakong, B. Fierz, D. M. Suter, Electrostatic properties of disordered regions control transcription factor search and pioneer activity. *Nat. Commun.* **17**, 2512 (2026).

22. M. Már, K. Nitsenko, P. O. Heidarsson, Multifunctional intrinsically disordered regions in transcription factors. *Chemistry* **29**, e202203369 (2023).

23. Y. Chen, C. Cattoglio, G. M. Dailey, Q. Zhu, R. Tjian, X. Darzacq, Mechanisms governing target search and binding dynamics of hypoxia-inducible factors. *eLife* **11**, e75064 (2022).

24. M. Iwafuchi, I. Cuesta, G. Donahue, N. Takenaka, A. B. Osipovich, M. A. Magnuson, H. Roder, S. H. Seeholzer, P. Santisteban, K. S. Zaret, Gene network transitions in embryos depend upon interactions between a pioneer transcription factor and core histones. *Nat. Genet.* **52**, 418–427 (2020).

25. Z. Wang, B. Wang, D. Niu, C. Yin, Y. Bi, C. Cattoglio, K. M. Loh, L. D. Lavis, H. Ge, W. Deng, Mesoscale chromatin confinement facilitates target search of pioneer transcription factors in live cells. *Nat. Struct. Mol. Biol.* **32**, 125–136 (2025).

26. M. V. Staller, Transcription factors perform a 2-step search of the nucleus. *Genetics* **222**, iyac111 (2022).

27. B. R. Sabari, A. Dall'Agnese, A. Boija, I. A. Klein, E. L. Coffey, K. Shrinivas, B. J. Abraham, N. M. Hannett, A. V. Zamudio, J. C. Manteiga, C. H. Li, Y. E. Guo, D. S. Day, J. Schuijers, E. Vasile, S. Malik, D. Hnisz, T. I. Lee, I. I. Cisse, R. G. Roeder, P. A. Sharp, A. K. Chakraborty,

R. A. Young, Coactivator condensation at super-enhancers links phase separation and gene control. *Science* **361**, eaar3958 (2018).

28. J. J. Ferrie, J. P. Karr, R. Tjian, X. Darzacq, "Structure"-function relationships in eukaryotic transcription factors: The role of intrinsically disordered regions in gene regulation. *Mol. Cell* **82**, 3970–3984 (2022).

29. S. Chong, C. Dugast-Darzacq, Z. Liu, P. Dong, G. M. Dailey, C. Cattoglio, A. Heckert, S. Banala, L. Lavis, X. Darzacq, R. Tjian, Imaging dynamic and selective low-complexity domain interactions that control gene transcription. *Science* **361**, eaar2555 (2018).

30. S. J. Bray, F. C. Kafatos, Developmental function of Elf-1: An essential transcription factor during embryogenesis in *Drosophila*. *Genes Dev.* **5**, 1672–1683 (1991).

31. A. E. Uv, E. J. Harrison, S. J. Bray, Tissue-specific splicing and functions of the *Drosophila* transcription factor Grainyhead. *Mol. Cell. Biol.* **17**, 6727–6735 (1997).

32. A. E. Uv, C. R. Thompson, S. J. Bray, The *Drosophila* tissue-specific factor Grainyhead contains novel DNA-binding and dimerization domains which are conserved in the human protein CP2. *Mol Cell Biol.* **14**, 4020–4031 (1994).

33. K. Venkatesan, H. R. McManus, C. C. Mello, T. F. Smith, U. Hansen, Functional conservation between members of an ancient duplicated transcription factor family, LSF/Grainyhead. *Nucleic Acids Res.* **31**, 4304–4316 (2003).

34. T. Wilanowski, A. Tuckfield, L. Cerruti, S. O'Connell, R. Saint, V. Parekh, J. Tao, J. M. Cunningham, S. M. Jane, A highly conserved novel family of mammalian developmental transcription factors related to *Drosophila* grainyhead. *Mech. Dev.* **114**, 37–50 (2002).

35. N. Traylor-Knowles, U. Hansen, T. Q. Dubuc, M. Q. Martindale, L. Kaufman, J. R. Finnerty, The evolutionary diversification of LSF and Grainyhead transcription factors preceded the radiation of basal animal lineages. *BMC Evol. Biol.* **10**, 101 (2010).

36. R. M. Reese, M. M. Harrison, E. T. Alarid, Grainyhead-like protein 2: The emerging role in hormone-dependent cancers and epigenetics. *Endocrinology* **160**, 1275–1288 (2019).

37. A. Paré, M. Kim, M. T. Juarez, S. Brody, W. McGinnis, The functions of grainy head-like proteins in animals and fungi and the evolution of apical extracellular barriers. *PLOS ONE* **7**, e36254 (2012).

38. K. A. Mace, J. C. Pearson, W. McGinnis, An epidermal barrier wound repair pathway in *Drosophila* is mediated by grainy head. *Science* **308**, 381–385 (2005).

39. J. Hemphälä, A. Uv, R. Cantera, S. Bray, C. Samakovlis, Grainy head controls apical membrane growth and tube elongation in response to Branchless/FGF signalling. *Development* **130**, 249–258 (2003).

40. C. Nüsslein-Volhard, E. Wieschaus, H. Kluding, Mutations affecting the pattern of the larval cuticle in *Drosophila melanogaster*. *Wilehm Roux Arch. Dev. Biol.* **193**, 267–282 (1984).

41. S. J. Bray, W. A. Johnson, J. Hirsh, U. Heberlein, R. Tjian, A cis-acting element and associated binding factor required for CNS expression of the *Drosophila melanogaster* dopa decarboxylase gene. *EMBO J.* **7**, 177–188 (1988).

42. B. D. Dynlacht, L. D. Attardi, A. Admon, M. Freeman, R. Tjian, Functional analysis of NTF-1, a developmentally regulated *Drosophila* transcription factor that binds neuronal cis elements. *Genes Dev.* **3**, 1677–1688 (1989).

43. S. B. Ting, T. Wilanowski, L. Cerruti, L.-L. Zhao, J. M. Cunningham, S. M. Jane, The identification and characterization of human Sister-of-Mammalian Grainyhead (SOM) expands the grainyhead-like family of developmental transcription factors. *Biochem. J.* **370**, 953–962 (2003).

44. A. Auden, J. Caddy, T. Wilanowski, S. B. Ting, J. M. Cunningham, S. M. Jane, Spatial and temporal expression of the *Grainyhead*-like transcription factor family during murine development. *Gene Expr. Patterns* **6**, 964–970 (2006).

45. S. Riethdorf, S. Frey, S. Santjer, M. Stoupiec, B. Otto, L. Riethdorf, C. Koop, W. Wilczak, R. Simon, G. Sauter, K. Pantel, V. Assmann, Diverse expression patterns of the EMT suppressor

grainyhead-like 2 (GRHL2) in normal and tumour tissues. *Int. J. Cancer* **138**, 949–963 (2016).

46. J. Jacobs, M. Atkins, K. Davie, H. Imrichova, L. Romanelli, V. Christiaens, G. Hulselmans, D. Potier, J. Wouters, I. I. Taskiran, G. Paciello, C. B. González-Blas, D. Koldere, S. Aibar, G. Halder, S. Aerts, The transcription factor Grainy head primes epithelial enhancers for spatiotemporal activation by displacing nucleosomes. *Nat. Genet.* **50**, 1011–1020 (2018).

47. M. Nevil, T. J. Gibson, C. Bartolutti, A. Iyengar, M. M. Harrison, Establishment of chromatin accessibility by the conserved transcription factor Grainy head is developmentally regulated. *Development* **147**, dev.185009 (2020).

48. A. F. Chen, A. J. Liu, R. Krishnakumar, J. W. Freimer, B. DeVeale, R. Blelloch, GRHL2-dependent enhancer switching maintains a pluripotent stem cell transcriptional subnetwork after exit from naive pluripotency. *Cell Stem Cell* **23**, 226–238.e4 (2018).

49. L. D. Attardi, R. Tjian, *Drosophila* tissue-specific transcription factor NTF-1 contains a novel isoleucine-rich activation motif. *Genes Dev.* **7**, 1341–1353 (1993).

50. H. A. Ertl, E. X. Bayala, M. A. Siddiq, P. J. Wittkopp, Divergence of Grainy head affects chromatin accessibility, gene expression, and embryonic viability in *Drosophila melanogaster*. bioRxiv 2024.04.07.588430 (2024).

51. F. Zhu, L. Farnung, E. Kaasinen, B. Sahu, Y. Yin, B. Wei, S. O. Dodonova, K. R. Nitta, E. Morgunova, M. Taipale, P. Cramer, J. Taipale, The interaction landscape between transcription factors and the nucleosome. *Nature* **562**, 76–81 (2018).

52. V. Ramalingam, X. Yu, B. D. Slaughter, J. R. Unruh, K. J. Brennan, A. Onyshchenko, J. J. Lange, M. Natarajan, M. Buck, J. Zeitlinger, Lola-I is a promoter pioneer factor that establishes de novo Pol II pausing during development. *Nat. Commun.* **14**, 5862 (2023).

53. P. T. Lowary, J. Widom, New DNA sequence rules for high affinity binding to histone octamer and sequence-directed nucleosome positioning1. *J. Mol. Biol.* **276**, 19–42 (1998).

54. R. S. Isaac, F. Jiang, J. A. Doudna, W. A. Lim, G. J. Narlikar, R. Almeida, Nucleosome breathing and remodeling constrain CRISPR-Cas9 function. *eLife* **5**, e13450 (2016).

55. M. G. Poirier, M. Bussiek, J. Langowski, J. Widom, Spontaneous access to DNA target sites in folded chromatin fibers. *J. Mol. Biol.* **379**, 772–786 (2008).

56. W. Antonin, H. Neumann, Chromosome condensation and decondensation during mitosis. *Curr. Opin. Cell Biol.* **40**, 15–22 (2016).

57. J. Gottesfeld, Mitotic repression of the transcriptional machinery. *Trends Biochem. Sci.* **22**, 197–202 (1997).

58. N. Naumova, M. Imakaev, G. Fudenberg, Y. Zhan, B. R. Lajoie, L. A. Mirny, J. Dekker, Organization of the mitotic chromosome. *Science* **342**, 948–953 (2013).

59. C. Deluz, E. T. Friman, D. Strebinger, A. Benke, M. Raccaud, A. Callegari, M. Leleu, S. Manley, D. M. Suter, A role for mitotic bookmarking of SOX2 in pluripotency and differentiation. *Genes Dev.* **30**, 2538–2550 (2016).

60. X. Liu, J. Shen, L. Xie, Z. Wei, C. Wong, Y. Li, X. Zheng, P. Li, Y. Song, Mitotic implantation of the transcription factor prospero via phase separation drives terminal neuronal differentiation. *Dev. Cell* **52**, 277–293.e8 (2020).

61. R. Silvério-Alves, I. Kurochkin, A. Rydström, C. Vazquez Echegaray, J. Haider, M. Nicholls, C. Rode, L. Thelaus, A. Y. Lindgren, A. G. Ferreira, R. Brandão, J. Larsson, M. F. T. R. De Bruijn, J. Martin-Gonzalez, C.-F. Pereira, GATA2 mitotic bookmarking is required for definitive haematopoiesis. *Nat. Commun.* **14**, 4645 (2023).

62. A. Chervova, A. Molliex, H. I. Baymaz, R.-X. Coux, T. Papadopoulou, F. Mueller, E. Hercul, D. Fournier, A. Dubois, N. Gaiani, P. Beli, N. Festuccia, P. Navarro, Mitotic bookmarking redundancy by nuclear receptors in pluripotent cells. *Nat. Struct. Mol. Biol.* **31**, 513–522 (2024).

63. N. Festuccia, A. Dubois, S. Vandormael-Pournin, E. Gallego Tejeda, A. Mouren, S. Bessonnard, F. Mueller, C. Proux, M. Cohen-Tannoudji, P. Navarro, Mitotic binding of Esrrb

marks key regulatory regions of the pluripotency network. *Nat. Cell Biol.* **18**, 1139–1148 (2016).

64. Y. Liu, B. Pelham-Webb, D. C. Di Giammartino, J. Li, D. Kim, K. Kita, N. Saiz, V. Garg, A. Doane, P. Giannakakou, A.-K. Hadjantonakis, O. Elemento, E. Apostolou, Widespread mitotic bookmarking by histone marks and transcription factors in pluripotent stem cells. *Cell Rep.* **19**, 1283–1293 (2017).

65. R. M. Price, M. A. Budzyński, J. Shen, J. E. Mitchell, J. Z. J. Kwan, S. S. Teves, Heat shock transcription factors demonstrate a distinct mode of interaction with mitotic chromosomes. *Nucleic Acids Res.* **51**, 5040–5055 (2023).

66. S. S. Teves, L. An, A. S. Hansen, L. Xie, X. Darzacq, R. Tjian, A dynamic mode of mitotic bookmarking by transcription factors. *eLife* **5**, e22280 (2016).

67. Q. Ming, Y. Roske, A. Schuetz, K. Walentin, I. Ibraimi, K. M. Schmidt-Ott, U. Heinemann, Structural basis of gene regulation by the Grainyhead/CP2 transcription factor family. *Nucleic Acids Res.* **46**, 2082–2095 (2018).

68. M. Nevil, E. R. Bondra, K. N. Schulz, T. Kaplan, M. M. Harrison, Stable binding of the conserved transcription factor grainy head to its target genes throughout *Drosophila melanogaster* development. *Genetics* **205**, 605–620 (2017).

69. J. T. Lis, Imaging *Drosophila* gene activation and polymerase pausing in vivo. *Nature* **450**, 198–202 (2007).

70. F. Persson, M. Lindén, C. Unoson, J. Elf, Extracting intracellular diffusive states and transition rates from single-molecule tracking data. *Nat. Methods* **10**, 265–269 (2013).

71. J. Liu, N. B. Perumal, C. J. Oldfield, E. W. Su, V. N. Uversky, A. K. Dunker, Intrinsic disorder in transcription factors. *Biochemistry* **45**, 6873–6888 (2006).

72. A. Katznelson, J. Zhang, G. Donahue, K. S. Zaret, Basis for lineage-determining pioneer factors targeting distinct repressed chromatin states. *Sci. Adv.* **12**, eadz7409 (2026).

73. J. Dufourt, A. Trullo, J. Hunter, C. Fernandez, J. Lazaro, M. Dejean, L. Morales, S. Nait-Amer, K. N. Schulz, M. M. Harrison, C. Favard, O. Radulescu, M. Lagha, Temporal control of gene expression by the pioneer factor Zelda through transient interactions in hubs. *Nat. Commun.* **9**, 5194 (2018).

74. M. A. F. Soares, D. S. Soares, V. Teixeira, A. Heskol, R. B. Bressan, S. M. Pollard, R. A. Oliveira, D. S. Castro, Hierarchical reactivation of transcription during mitosis-to-G1 transition by Brn2 and Ascl1 in neural stem cells. *Genes Dev.* **35**, 1020–1034 (2021).

75. A. L. Sanborn, B. T. Yeh, J. T. Feigerle, C. V. Hao, R. J. Townshend, E. Lieberman Aiden, R. O. Dror, R. D. Kornberg, Simple biochemical features underlie transcriptional activation domain diversity and dynamic, fuzzy binding to Mediator. *eLife* **10**, e68068 (2021).

76. A. J. Ruthenburg, H. Li, T. A. Milne, S. Dewell, R. K. McGinty, M. Yuen, B. Ueberheide, Y. Dou, T. W. Muir, D. J. Patel, C. D. Allis, Recognition of a mononucleosomal histone modification pattern by BPTF via multivalent interactions. *Cell* **145**, 692–706 (2011).

77. M. M. Harrison, M. R. Botchan, T. W. Cline, Grainyhead and Zelda compete for binding to the promoters of the earliest-expressed *Drosophila* genes. *Dev. Biol.* **345**, 248–255 (2010).

78. D. C. Hamm, E. R. Bondra, M. M. Harrison, Transcriptional activation is a conserved feature of the early embryonic factor Zelda that requires a cluster of four zinc fingers for DNA binding and a low-complexity activation domain. *J. Biol. Chem.* **290**, 3508–3518 (2015).

79. Peptide Nexus, Scramble Peptide or Protein Sequence; https://peptidenexus.com/article/sequence-scrambler.

80. R. D. Vale, J. A. Spudich, E. R. Griffis, Dynamics of myosin, microtubules, and Kinesin-6 at the cortex during cytokinesis in *Drosophila* S2 cells. *J. Cell Biol.* **186**, 727–738 (2009).

81. F. J. DeHaro-Arbona, C. Roussos, S. Baloul, J. Townson, M. J. Gómez Lamarca, S. Bray, Dynamic modes of Notch transcription hubs conferring memory and stochastic activation revealed by live imaging the co-activator Mastermind. *eLife* **12**, RP92083 (2024).

82. M. J. Gomez-Lamarca, J. Falo-Sanjuan, R. Stojnic, S. Abdul Rehman, L. Muresan, M. L. Jones, Z. Pillidge, G. Cerda-Moya, Z. Yuan, S. Baloul, P. Valenti, K. Bystricky, F. Payre, K. O'Holleran, R. Kovall, S. J. Bray, Activation of the Notch signaling pathway in vivo elicits changes in CSL nuclear dynamics. *Dev. Cell* **44**, 611–623.e7 (2018).

83. S. Baloul, C. Roussos, M. Gomez-Lamarca, L. Muresan, S. Bray, Changes in searching behaviour of CSL transcription complexes in Notch active conditions. *Life Sci. Alliance* **7**, e202302336 (2023).

84. M. Ovesný, P. Křížek, J. Borkovec, Z. Švindrych, G. M. Hagen, ThunderSTORM: A comprehensive ImageJ plug-in for PALM and STORM data analysis and super-resolution imaging. *Bioinformatics* **30**, 2389–2390 (2014).

85. N. Chenouard, I. Bloch, J.-C. Olivo-Marin, Multiple hypothesis tracking for cluttered biological image sequences. *IEEE Trans. Pattern Anal. Mach. Intell.* **35**, 2736–3750 (2013).

86. A. S. Hansen, A. Amitai, C. Cattoglio, R. Tjian, X. Darzacq, Guided nuclear exploration increases CTCF target search efficiency. *Nat. Chem. Biol.* **16**, 257–266 (2020).

87. R. J. Emenecker, D. Griffith, A. S. Holehouse, Metapredict: A fast, accurate, and easy-to-use predictor of consensus disorder and structure. *Biophys. J.* **120**, 4312–4319 (2021).

88. A. S. Holehouse, R. K. Das, J. N. Ahad, M. O. G. Richardson, R. V. Pappu, CIDER: Resources to analyze sequence-ensemble relationships of intrinsically disordered proteins. *Biophys. J.* **112**, 16–21 (2017).

89. P. Machanick, T. L. Bailey, MEME-ChIP: Motif analysis of large DNA datasets. *Bioinformatics* **27**, 1696–1697 (2011).
